# CHN1 as a potential predictive genetic biomarker for atopic dermatitis-related depression

**DOI:** 10.3389/fimmu.2025.1677275

**Published:** 2025-11-17

**Authors:** Yifei Wang, Yuqing Liu, Miao Chen, Danping Liu, Chen Shen

**Affiliations:** 1 Department of Dermatology, Union Hospital, Tongji Medical College, Huazhong University of Science and Technology, Wuhan, China; 2 Department of Dermatology, Zhongnan Hospital of Wuhan University, Wuhan University, Wuhan, China

**Keywords:** atopic dermatitis (AD), depression, CHN1, neuro-immune mechanism, comorbidity, gene

## Abstract

**Introduction:**

The comorbidity of atopic dermatitis (AD) and depression has garnered increased attention in recent years, yet the immunopathological mechanisms underlying this connection remain unclear. To bridge this gap, the study aimed to uncover the immune regulatory networks and identify key genetic markers involved in the comorbidity of depression in AD.

**Methods:**

We performed RNA sequencing on peripheral blood mononuclear cells (PBMCs) collected from 20 AD patients with and without depression. By integrating bioinformatics analyses with machine learning, we conducted weighted gene co-expression network analysis (WGCNA), functional enrichment analysis, and employed machine learning models of least absolute shrinkage and selection operator (LASSO) and support vector machine-recursive feature elimination (SVM-RFE). Additionally, validation was carried out in an independent cohort of 20 participants to confirm the expression of the identified potential pivotal gene.

**Results:**

A total of 394 differentially expressed genes (DEGs) were identified in AD patients with depression as compared to those non-depressed counterparts. Weighted gene co-expression network analysis (WGCNA) pinpointed a pink module encompassing 83 genes strongly linked to depressive symptoms. Functional enrichment analysis highlighted biological processes related to neurotransmitter uptake and the negative regulation of T-helper (Th) 17 cell differentiation. Furthermore, machine learning models of least absolute shrinkage and selection operator (LASSO) and support vector machine-recursive feature elimination (SVM-RFE) consistently identified CHN1 as a potential pivotal gene upregulated in AD patients with depression. The expression level of CHN1 demonstrated positive correlation with Th2 and Th17 cytokine signatures, as well as with the Hospital Anxiety and Depression Scale-Depression (HADS-D) score, and the Eczema Area and Severity Index (EASI). Validation in an independent cohort of 20 participants confirmed the significant upregulation of CHN1 in depressed AD patients.

**Discussion:**

Together, these findings reveal previously unrecognized immunoinflammatory axis underlying AD-associated depression, and shed light on CHN1 as a potential molecular bridge connecting peripheral inflammation and neuropsychiatric manifestations.

## 1 Introduction

Atopic dermatitis (AD), the most common chronic inflammatory skin disease, affects up to 20% of children and 10% of adults, imposing significant burdens to patients’ quality of life and mental health (1–3). Patients with AD are commonly characterized by eczematous rashes, diffuse xerosis, intense pruritus, with *Staphylococcus aureus* infections (4). Numerous clinical and epidemiological studies have established a significant association between AD and various psychiatric comorbidities (5–10). Such mental comorbidity not only exacerbates individual suffering, but also imposes substantial socioeconomic burdens, particularly in the long-term management of chronic diseases (11). Among these psychiatric disorders, depression is one of the most prevalent, manifesting as persistent low mood, fatigue, and cognitive impairment (12). Existing research data have revealed a dose-response relationship between AD severity and the risk of developing depression (7).In one cohort of 695 patients, 14.68% exhibited moderate-to-severe depressive symptoms, which closely fluctuated with the severity of AD (13). Despite these observations, the central mechanisms linking AD to depression comorbidity remains poorly defined, impeding progress in early identification and targeted intervention.

Chronic peripheral inflammation is thought to play a critical role in triggering central neuropsychiatric disorders (14). Mounting evidence indicates that peripheral immune dysregulation contributes to the onset and maintenance of AD-associated depressive symptoms (15, 16). In AD, a pronounced T-helper 2 (Th2) cell response, marked by the secretion of Interleukin (IL)-4, IL-5, IL-33, and IL-13, contributes to the formation of a complex cytokine milieu (17–20). This environment can activate peripheral sensory nerves that transmit chronic itching signals to the central nervous system (CNS), thereby affecting neural circuits responsible for pruritus and mood regulation (21). Additionally, peripheral inflammatory factors can compromise blood-brain barrier integrity, and activate microglia and astrocytes, which exacerbates neuroinflammatory cascades, ultimately contributing to depressive phenotypes (22–24). However, researches on these mechanisms remain fragmented, and the interplay between the peripheral immune networks and central neuropsychiatric comorbidities has yet to be systemically explored.

Peripheral blood mononuclear cells (PBMCs) from patients with AD exhibit distinct transcriptional signatures that mirror their systemic inflammation activation, making them an accessible model for studying disease-related inflammation. Integrating transcriptomic data from PBMCs with machine learning approaches allowing comprehensive analysis of high-dimensional transcriptomic datasets, can identify key genes, construct immune regulatory networks, and pinpoint potential biomarkers with unprecedented accuracy (25, 26).

**Figure 1:**
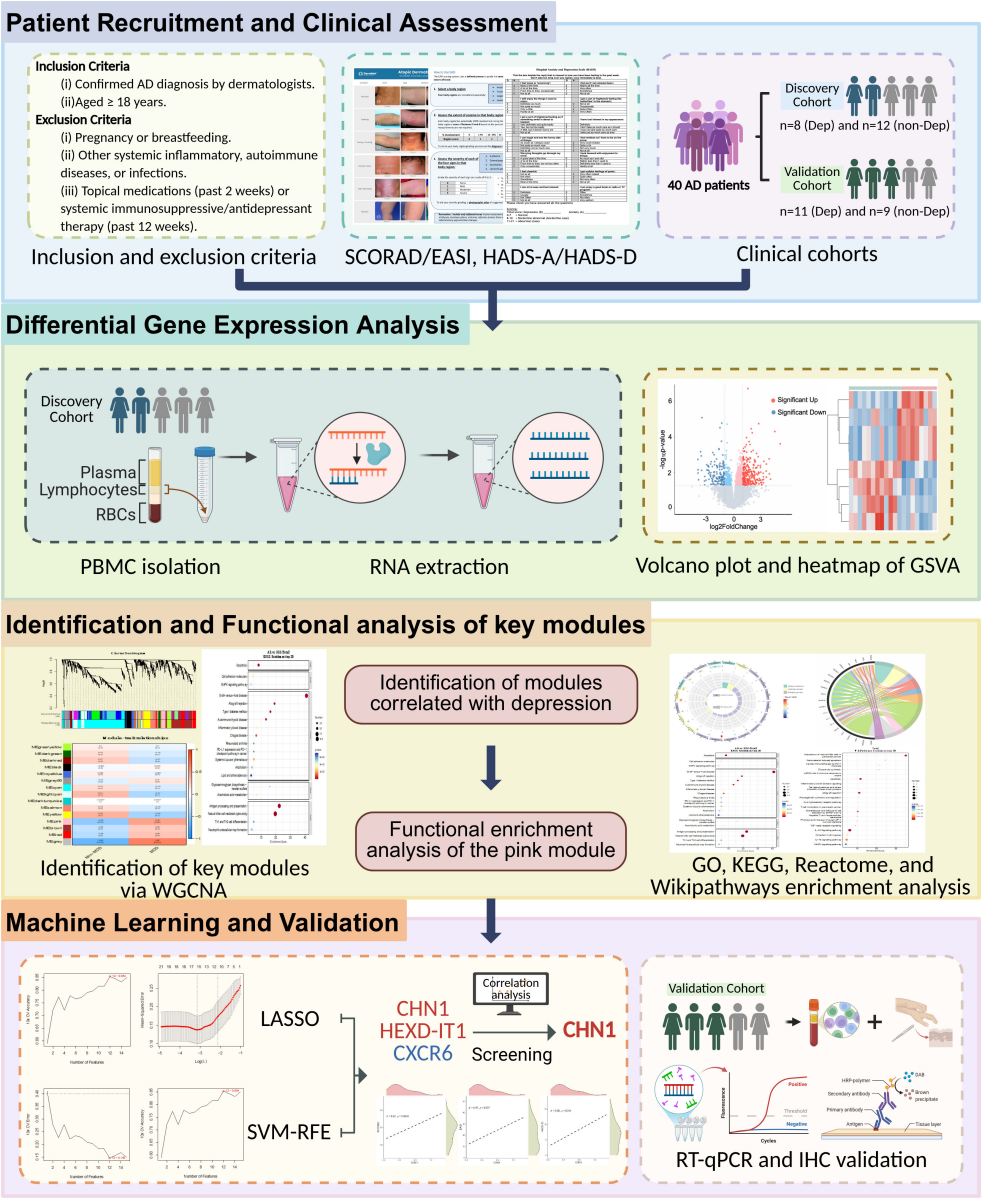
Analytical workflow of the study.

In this study, we conducted transcriptomic analyses of PBMCs from AD patients with or without depression, and employed integrative bioinformatics and machine learning approaches to systemically delineate the molecular mechanisms underlying the depression comorbidity. We further validated the key genes and examined their associations with immune pathways, the disease severity, and depressive symptom scores. These findings provide new insights into the inflammation-depression axis in AD and lay the groundwork for precision diagnostics and targeted interventions in AD-related neuropsychiatric comorbidities. The overall analytical workflow is illustrated in **Figure 1**.

## 2 Materials and methods

### 2.1 Participants

The study enrolled 40 adult patients diagnosed with AD at Wuhan Union Hospital. The inclusion criteria were: (i) a confirmed diagnosis of AD, established by dermatologists according to the guidelines of the American Academy of Allergy, Asthma and Immunology (27), and (ii) being 18 years of age or older. The exclusion criteria included: (i) pregnancy or breastfeeding; (ii) the presence of other known systemic inflammatory diseases, autoimmune disorders, or infections; and (iii) the use of any topical medications within the previous 2 weeks or systemic immunosuppressive or antidepressant therapy within the previous 12 weeks. Among the participants, 19 met the diagnostic criteria for depressive disorder, based on the psychiatrists’ application using the Fifth Edition of the Diagnostic and Statistical Manual of Mental Disorders (DSM-5), detailed in **Supplementary Table 1** (28). All participants provided written informed consent. The study was approved by the Ethics Committee of Wuhan Union Hospital (protocol No. UHCT240584), and conducted in accordance with the Declaration of Helsinki.

### 2.2 Assessment of clinical characteristics

Clinical characteristics were evaluated including the SCORing of Atopic Dermatitis (SCORAD), Eczema Area and Severity Index (EASI), Hospital Anxiety and Depression Scale-Anxiety (HADS-A) subscale, and HADS-Depression (HADS-D) subscale. SCORAD assesses objective signs (lesion area and severity, 0-80), subjective symptoms (pruritus and sleep disturbance, 0-20), and a visual analog scale (VAS, 0-10) for overall severity, with a total score ranging from 0 to 100 (29). EASI focuses on objective evaluation, including lesion area (weighted by body regions, 0-6) and severity (erythema, infiltration/papulation, excoriation, lichenification, 0-72), with a total score of 0-72 (30). Each HADS subscale uses seven 0–3 Likert items to yield anxiety and depression scores from 0 to 21, with ≥ 8 indicating clinically significant symptoms (31, 32).

### 2.3 PBMC isolation, RNA extraction and library preparation

Peripheral blood samples were collected from participants in heparin-coated tubes. PBMCs were isolated using Lymphocyte Separation Medium (Corning, Manassas, VA) through density-gradient centrifugation. Cell counts were determined using the Cellometer Auto 2000 (Nexcelom, Lawrence, MA), and cells were cultured at a density of 2 × 10^6^ cells/ml. Total RNA was extracted from PBMCs using TRIzol reagent (Invitrogen, Carlsbad, CA), and RNA quality was assessed using the NanoDrop ND-1000 spectrophotometer (ThermoFisher Scientific, Waltham, MA). Strand-specific RNA-seq libraries were subsequently prepared with the VAHTS Universal V6 RNA-seq Library Prep Kit following the manufacturer’s instructions.

### 2.4 RNA sequencing and differential gene expression analysis

Libraries were sequenced on an Illumina NovaSeq 6000 platform, generating ~60 million 150-bp paired-end reads per sample. After adapter trimming and quality filtering with fastp, an average of 57.2 million clean reads (Q30 ≥ 93%) remained. These were aligned to the GRCh38 reference genome with HISAT2 (unique-mapping rate 91.8%), and gene-level abundances were quantified as both FPKM (StringTie) and raw counts (HTSeq-count). All raw data have been deposited in the GEO database (accession GSE307177).

Differential gene expression analysis comparing AD patients with and without comorbid depression was performed using the DESeq2 packages in R software. Raw read counts were normalized with the built-in median-of-ratios method, and baseMean values were used to estimate average expression levels. Fold changes were calculated from the normalized counts, and significance was assessed by a negative-binomial Wald test implemented in DESeq2 (33). Differentially expressed genes (DEGs) were identified based on the criteria of adjusted *P* < 0.05 (Benjamini–Hochberg) and |log_2_ fold change (log_2_FC) | > 1 (34). Volcano plots and heatmaps were generated to visualize DEGs using the ‘heatmap’ and ‘ggplot2’ packages.

### 2.5 Weighted gene co-expression network analysis

WGCNA was performed using the WGCNA R package to identify co-expression gene modules, potentially related to depression in AD. A soft thresholding was applied to establish a scale-free network topology. Modules were identified using hierarchical clustering with a dynamic tree-cut algorithm. Module eigengenes (MEs) were calculated to represent the first principal component of each module, and correlation analysis between MEs and clinical traits was performed to identify depression-related modules (35).

### 2.6 Pathway enrichment analysis

Pathway enrichment in AD-related depression datasets was evaluated using Gene Set Variation Analysis (GSVA). All hallmark gene sets were obtained from the Molecular Signature Database (MSigDB). An adjusted *P*-value < 0.05 was considered statistically significant after Benjamini and Hochberg correction (36).

### 2.7 Functional enrichment analysis

Functional enrichment analysis of DEGs was performed using the Gene Ontology (GO) plot, ReactomePA and clusterProfiler packages in R. GO functional annotation, Kyoto Encyclopedia of Genes and Genomes (KEGG) pathway analyses, Reactome and WikiPathways enrichment analysis were conducted to explore the biological roles of hub genes. The annotation terms with *P*-value < 0.05 were considered significantly enriched, and results were visualized in a bubble diagram and heat map (37, 38).

### 2.8 Machine learning for key genes

Two machine learning algorithms were employed to identify key genes associated with depression in AD patients. Least Absolute Shrinkage and Selection Operator (LASSO) was performed (glmnet R package) to select a subset of genes with the highest predictive power. The model was fitted with 10-fold cross-validation and nlambda = 100, and the λ value was selected to minimize the mean-squared error, which represents the optimal LASSO fit and minimizes the cross-validation error. The gene count at this point is taken as the number of disease-signature genes. Support Vector Machine Recursive Feature Elimination (SVM-RFE) was conducted (caret package) to rank genes based on their importance in predicting depression. Ten-fold cross-validation (with a fixed random seed) was applied to the SVM-RFE pipeline. The average rank of each feature across all folds was computed to determine the optimal feature subset and used plotting functions to visualize the trends in both generalization error and classification accuracy across gene numbers. A Venn diagram was constructed to identify overlapping genes selected by both methods (38–40).

### 2.9 Receiver operating characteristic curve analysis

ROC curve analysis was performed in R to assess the diagnostic performance of key genes. The area under the curve (AUC) was calculated to validate the diagnostic value of key genes.

### 2.10 Quantitative reverse transcription-polymerase chain reaction

Total RNA extracted from participants’ PBMCs was reverse—transcribed into cDNA. Following cDNA synthesis, RT-qPCR was conducted using CHN1-specific primers (**Table 1**), and the relative expression levels were quantified and statistically analyzed using standard ΔΔCt methods and analyzed statistically.

**Table 1:** The primer sequences of CHN1 and ACTB.

Gene	Forward primer (5’ to 3’)	Reverse primer (5’ to 3’)
CHN1	CCTGTACTTGCGAGGTGGAA	CCAAAGTGTAGGTCCCTGGC
ACTB	GCCGCCAGCTCACCAT	GCTGACTGTGAACTCCCTCC

CHN1, chimerin-1; ACTB, β- Actin.

### 2.11 Immunohistochemistry staining

Skin lesion samples all collected from the limbs were used for IHC. Slides were immunostained for CHN1, and the extent of immunostaining was reviewed and scored by two independent dermatopathologists, blinded to clinical details. Immunostaining scores were calculated by multiplying the percentage of positive cells by staining intensity.

### 2.12 Statistical analysis

All statistical analyses were performed using R software. Continuous variables were compared using Student’s t-test, and categorical variables were compared using chi-square or Fisher’s exact test. Correlation analysis was performed using Pearson’s correlation coefficients, depending on the normality of the data. A two-tailed *P*-value <0.05 was considered statistically significant.

## 3 Results

### 3.1 Participants characteristics

Skin lesion and PBMC samples were collected from two independent participant cohorts. The discovery cohort included 8 AD patients with depression and 12 without, whose PBMCs were subjected to RNA sequencing and DEG analysis. The validation cohort consisted of 11 AD patients with depression and 9 without, providing clinical samples to validate the identified DEGs (**Table 2**). All patients met the established diagnostic criteria. No statistically significant differences were observed in demographics or skin severity scores (SCORAD, EASI) between groups. However, anxiety and depression scale (HADS-A, HADS-D) scores were substantially higher in AD patients with depression relative to those without.

**Table 2:** Characteristics of study participants and data on disease characteristics and comorbid depression.

Characteristic	Discover set in AD		*P*-value	Validation set in AD		*P*-value
	Depression n=8	No depression n=12		Depression n=11	No depression n=9	
Age (years), mean ± SD	49.00 ± 18.89	36.08 ± 18.53	0.147	38.91 ± 16.71	40.11 ± 17.00	0.876
Female, n (%)	3 (37.50)	4 (33.33)	> 0.999	5 (45.45)	3 (33.33)	> 0.999
Body mass index (kg/m^2^), mean ± SD	23.04 ± 1.74	21.59 ± 1.63	0.200	23.26 ± 1.98	22.86 ± 1.52	0.725
Disease duration (years), mean ± SD	7.25 ± 5.90	5.88 ± 5.53	0.602	7.73 ± 6.66	8.33 ± 7.00	0.845
SCORAD, mean ± SD	62.24 ± 17.86	48.28 ± 20.10	0.130	63.71 ± 18.23	58.29 ± 20.08	0.838
EASI, mean ± SD	22.13 ± 10.83	17.28 ± 13.51	0.408	37.85 ± 15.51	34.30 ± 18.88	0.837
HADS-A, mean ± SD	11.50 ± 4.50	5.42 ± 3.90	0.005	15.27 ± 2.37	4.44 ± 3.28	< 0.0001
HADS-D, mean ± SD	13.00 ± 3.07	3.33 ± 2.61	< 0.001	14.36 ± 2.91	3.33 ± 2.50	< 0.0001

AD, atopic dermatitis; SD, standard deviation; SCORAD, scoring atopic dermatitis; EASI, eczema area and severity index; HADS-A, Hospital Anxiety and Depression Scale-Anxiety; HADS-D, Hospital Anxiety and Depression Scale-Depression.

### 3.2 DEG analysis of AD patients with and without depression

**Figure 2:**
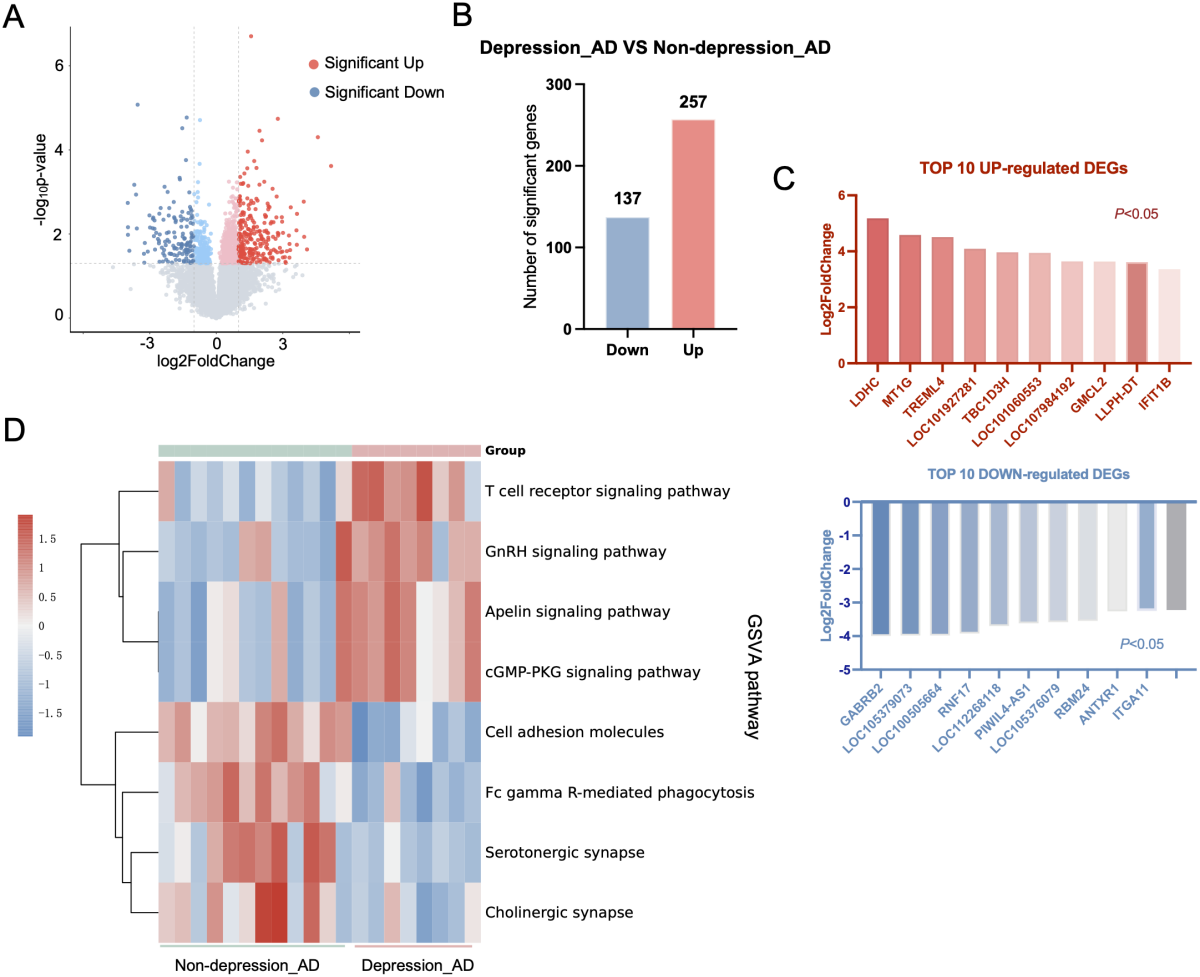
Baseline gene expression analysis in AD patients with and without depression. **(A)** Volcano plot showing DEGs in depressed AD patients compared with non-depressed counterparts. Blue dots represent downregulated genes and red plots represent upregulated genes. **(B)** Bar chart showing the number of down- and up-regulated DEGs in AD patients with depression. **(C)** Top 10 up- and down-regulated DEGs ranked by fold change and Log2FC (*P* < 0.05). **(D)** Heatmap displaying GSVA enrichment analysis results, highlighting key pathways associated with depression in AD patients.

To identify the molecular features associated with depression in AD, we analyzed PBMC transcriptomes from both groups. Under the criteria of *P*-adjustment <0.05 and log_2_ FC >1, a total of 394 DEGs were identified, 257 up-regulated and 137 down-regulated genes, in depressed AD patients compared with non-depressed counterparts. These genes were visualized using a volcano plot (**Figures 2A, B**), and the top 10 up- and down-regulated DEGs were visualized using a volcano plot (**Figure 2C**). GSVA analysis revealed that the gene profiles of AD patients with depression were mainly enriched in the T-cell receptor signaling pathway, GnRH signaling pathway, Apelin signaling pathway, and the cGMP-PKG signaling pathway, etc (**Figure 2D**).

### 3.3 Identification of key modules via WGCNA

**Figure 3:**
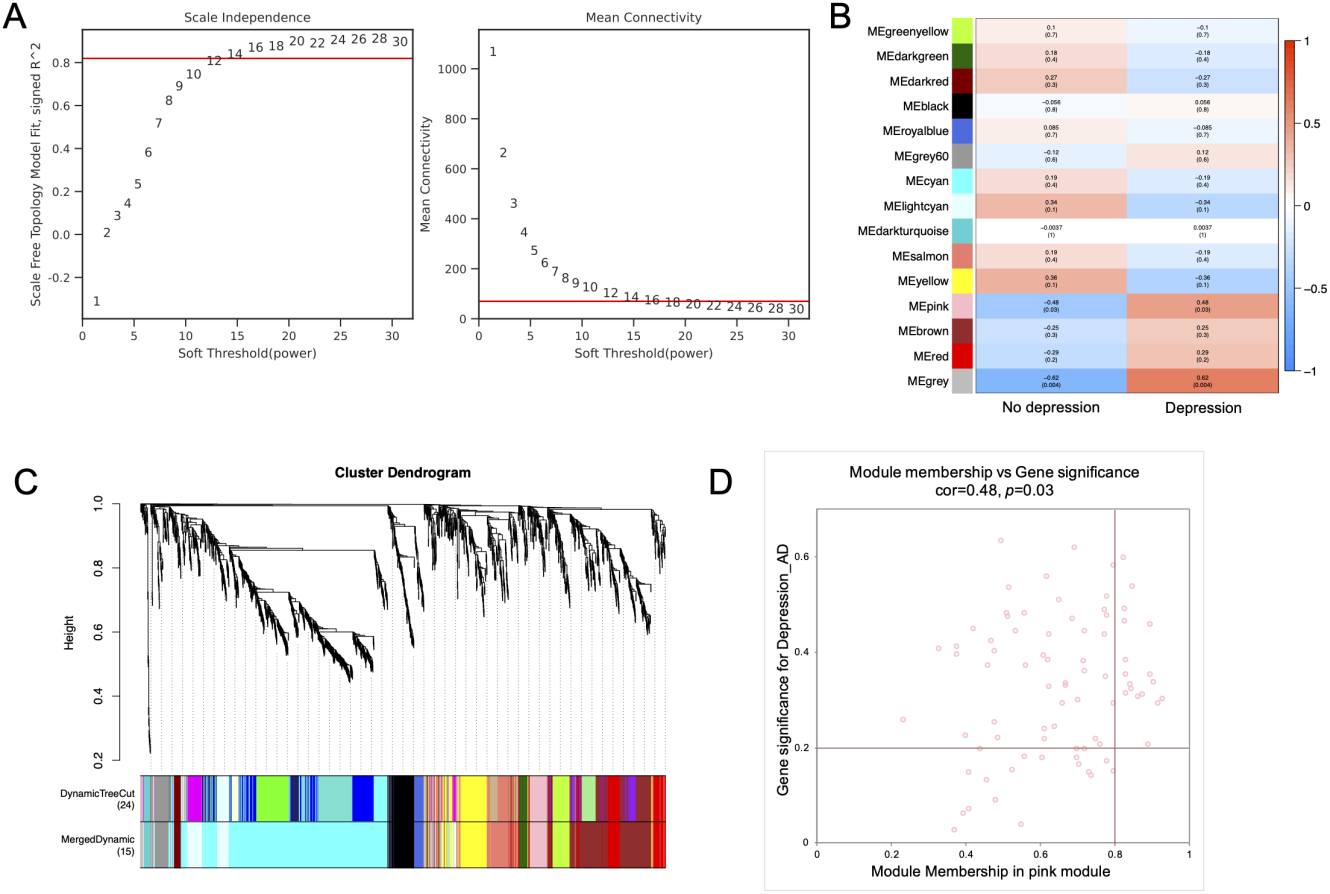
Identification of modules highly correlated with depression in AD patients. **(A)** Topology analysis and mean connectivity assessment across a range of soft threshold powers. **(B)** Heatmap illustrating the relevance of various modules with depression. **(C)** Dendrogram of module clustering, with distinct module colors representing different gene module. **(D)** The pink module was identified as the module most significantly correlated with depression in AD. The scatter plot presenting the correlation between module membership and gene significance within the module.

WGCNA analysis was constructed to screen out the core genes associated with depression in AD patients As shown in **Figure 3A**, with a soft threshold to 14 (R^2^>0.82) and a high average connectivity, 15 modules were identified after merging the strongly associated modules using a height cutoff of 0.25. **Supplementary Figure 1** further validated the test results at power 14 and showed the Topological Overlap Matrix (TOM). The module clustering dendrogram showed the primed and merged modules in AD patients with depression (**Figure 3B**). Correlation analysis of ME values and clinical manifestations revealed a significant association between the pink module and depression (r = 0.48, *P* < 0.05), while a negative correlation was observed with non-depression (r = −0.48, *P* < 0.05) (**Figure 3C**). Genes in the grey module, which lack module assignment, were excluded from further analysis. The scatter plot analysis confirmed the strong association between the pink module and depression (**Figure 3D**), and 83 genes within this module were selected for further analysis.

### 3.4 Functional enrichment analysis of critical module genes

**Figure 4:**
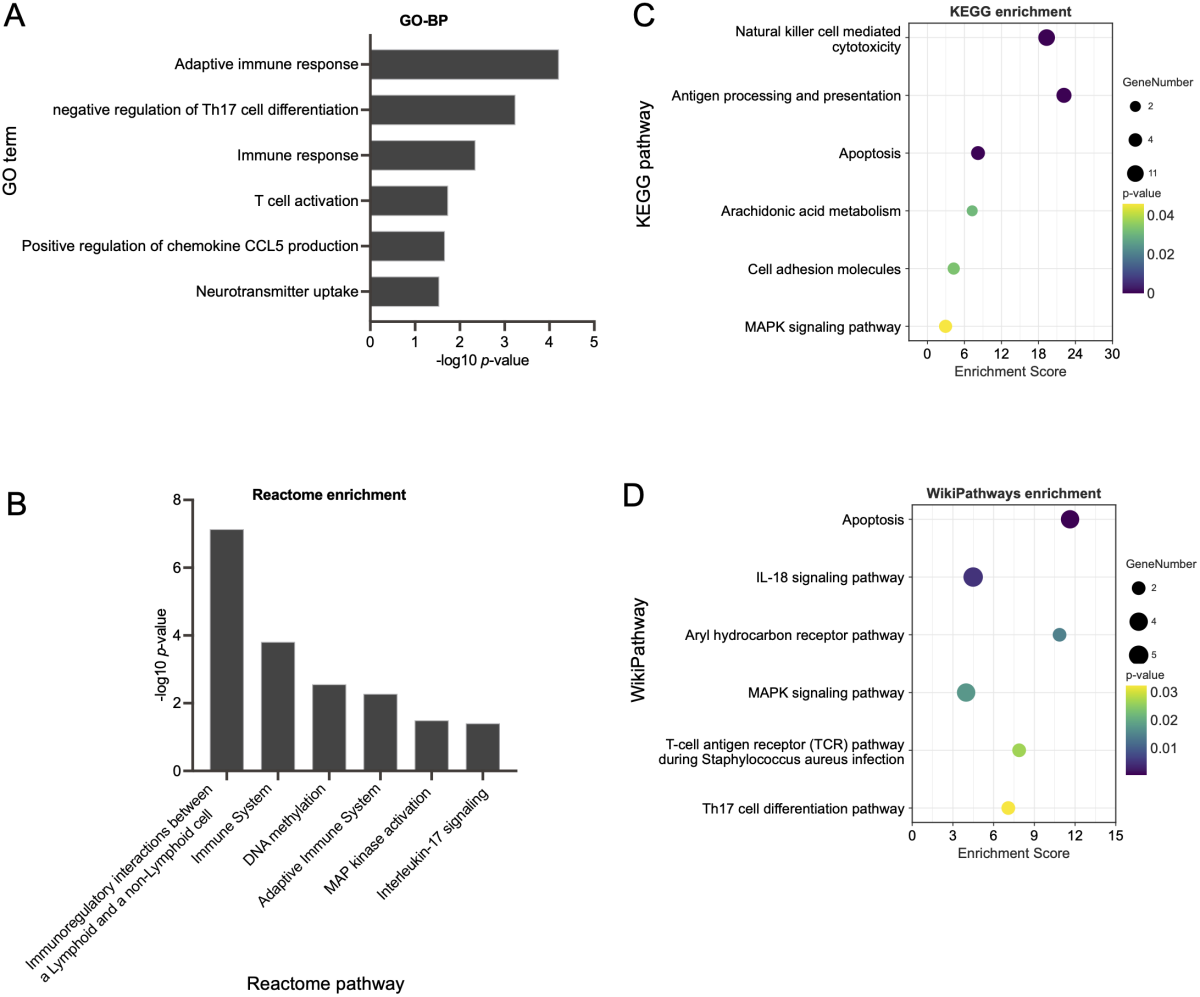
Functional enrichment analysis of genes within the pink module. **(A)** GO enrichment analysis in biological processes (BP), **(B)** KEGG pathway enrichment analysis, **(C)** Reactome enrichment analysis, and **(D)** WikiPathways enrichment analysis identifying key signaling pathways within the pink module that are associated with AD patients with depression.

Functional enrichment analysis within the pink module uncovered the biological roles of DEGs, which most reflected the key signaling pathways activated in AD patients with depression. GO analysis revealed associations with neural and immune pathways, including adaptive immune response, negative regulation of Th17 cell differentiation, T cell activation, positive regulation of chemokine CCL5 production and neurotransmitter uptake (**Figure 4A**). KEGG enrichment analysis highlighted pathways such as antigen processing and presentation, natural killer cell-mediated cytotoxicity, apoptosis, arachidonic acid metabolism, and the MAPK signaling pathway (**Figure 4B**). Reactome pathway analysis showed enrichment in immune system interactions, immunoregulatory interactions between a lymphoid and a non-lymphoid cell, IL-17 signaling, etc. (**Figure 4C**). WikiPathways analysis emphasized high enrichment scores in IL-18 signaling pathway, aryl hydrocarbon receptor pathway, T-cell antigen receptor pathway during staphylococcus aureus infection, and Th17 cell differentiation pathway (**Figure 4D**).

### 3.5 Identification of key genes by machine learning

**Figure 5:**
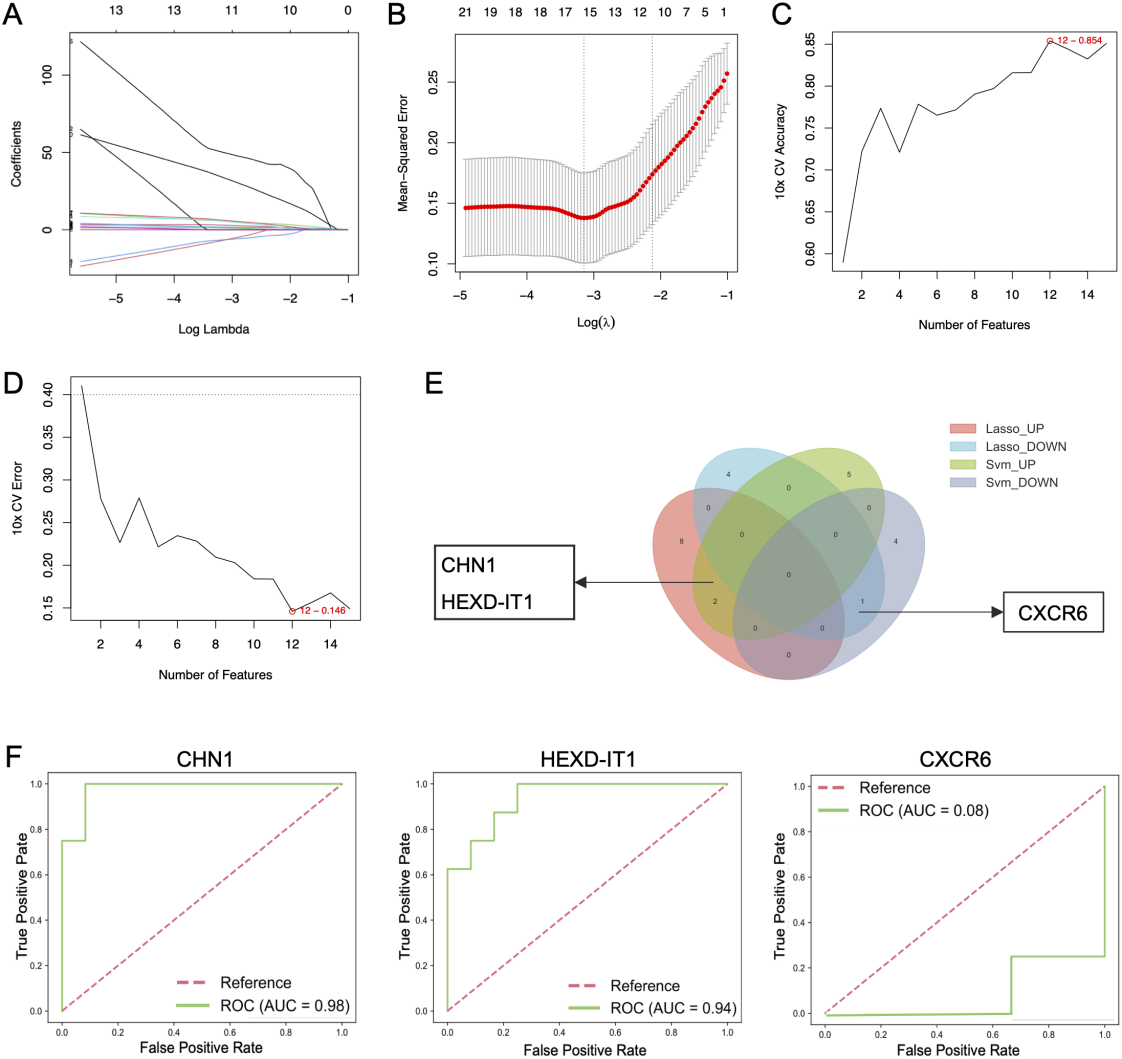
Identification of key genes in AD patients with and without depression using machine learning methods. **(A, B)** Identification of hub genes in AD patients with depression using LASSO regression analysis, and **(C, D)** SVM-RFE algorithms. **(E)** Venn diagram screening overlapping hub genes identified by both LASSO regression analysis and SVM-RFE. **(F)** ROC curve analysis of core genes.

Key genes were identified using LASSO regression analysis and SVM-RFE algorithms. Through the rigorous process of LASSO analysis (10-fold CV, mean AUC = 0.81 ± 0.3), 15 feature genes (10 up-regulated and 5 down-regulated) were yielded (**Figures 5A, B**), while SVM-RFE (10-fold CV, AUC = 0.75 ± 0.17) identified 12 genes (7 upregulated and 5 down-regulated) (**Figures 5C, D**). The Venn diagram revealed the overlap of key genes identified by the two machine learning methods discussed above (**Figure 5E**). From the pool of identified genes, *CHN1* and *HEXD-IT1* emerged as upregulated in AD patients with comorbid depression, whereas *CXCR6* was pinpointed as a downregulated gene. ROC analysis illustrated revealed that *CHN1* and *HEXD-IT1* exhibited high AUC values (> 0.8), indicating a strong association of these two genes with AD-related depression (**Figure 5F**).

### 3.6 Immune-related interactions of key genes

**Figure 6:**
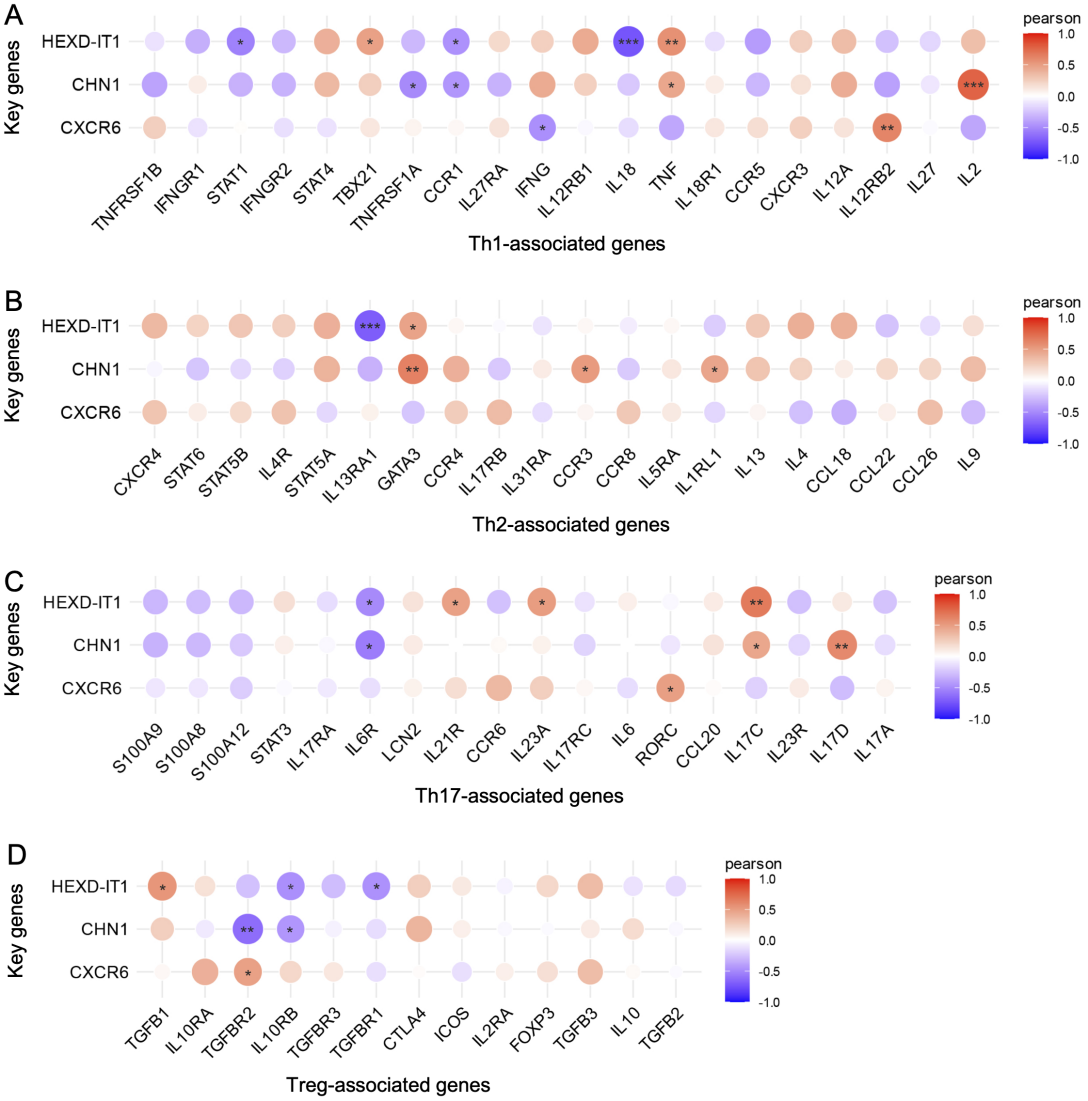
Relationship between immune-related active molecules and key genes. Correlation analysis of key genes with members of the **(A)** Th1, **(B)** Th2, **(C)** Th17, and **(D)** Treg family members. *p < 0.05, *p < 0.01, **p < 0.01, ***p < 0.001.

To delve deeper into the immunological functions of key genes, we investigated their connections with activators across the four principal immune cell families. In the Th1 lineage, IL2 exhibited a substantial positive linkage with *CHN1* (**Figure 6A**). In the Th2 family, markers such as *GATA3*, *CCR3*, and *IL1RL1* demonstrated significant associations with *CHN1* (**Figure 6B**). Within the Th17 subset, *IL17* and *IL23* were strongly correlated with the elevated expression of *CHN1* and *HEXD-IT1*, underscoring their significance within the immune response landscape (**Figure 6C**). Conversely, no significant associations were detected between *CXCR6* and the Th1, Th2 or Th17 families. However, in the Treg family, *TGFBR2*, *IL10RB*, and *TGFB1* were negatively correlated with *CHN1* and *HEXD-IT1* but positively correlated with *CXCR6* (**Figure 6D**). These findings suggest that the Th2 and Th17 pathways may drive the comorbidity of anxiety and depression in AD, while Treg family might exert a counter-regulatory effect.

### 3.7 Correlations between key genes and clinical features

**Figure 7:**
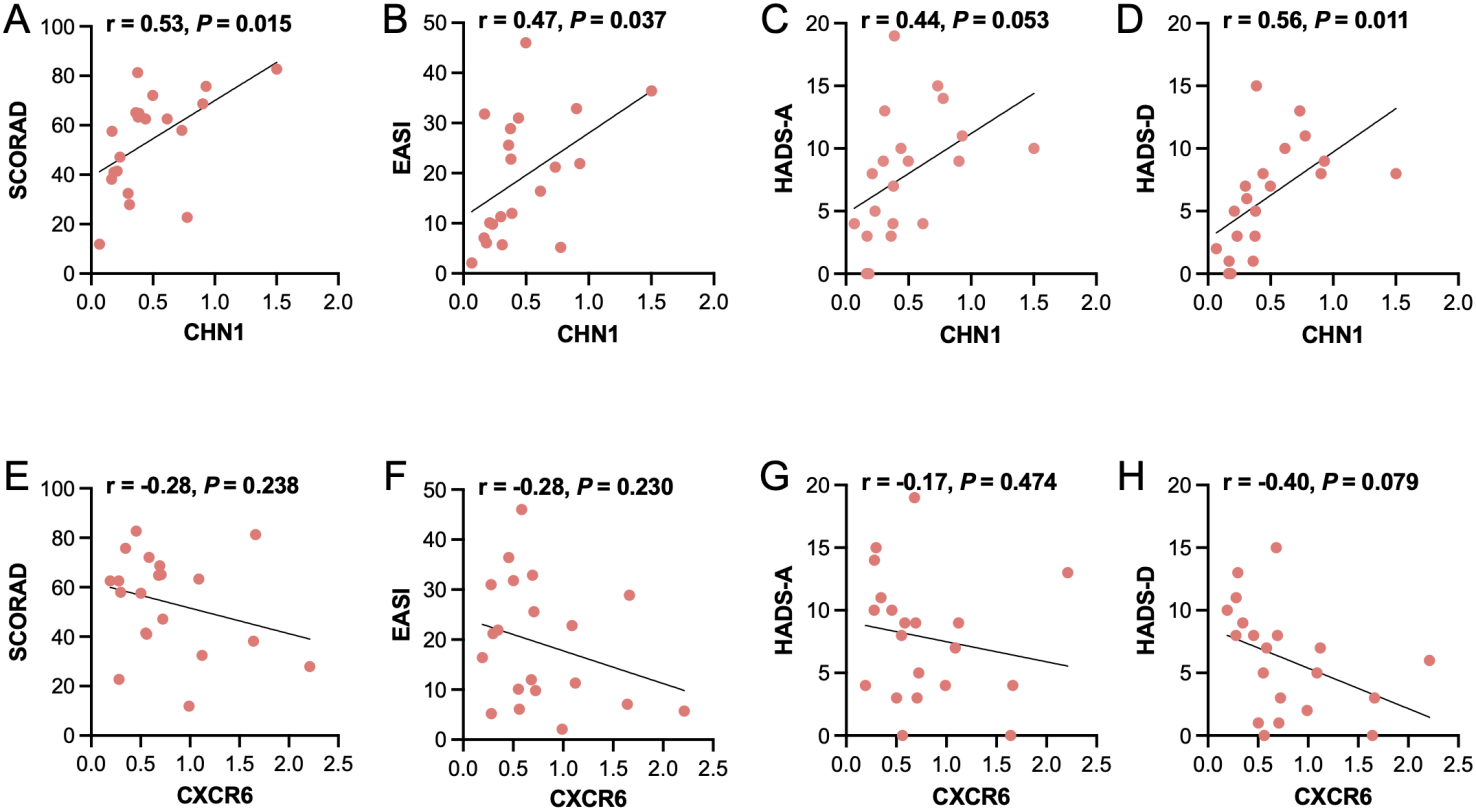
Correlation between CHN1 and CXCR6 expression and clinical characteristics. Pearson correlation analysis of CHN1 mRNA expression levels with clinical characteristics, including **(A)** SCORAD, **(B)** EASI, **(C)** HADS-A, and **(D)** HADS-D. Correlation of CXCR6 mRNA expression levels with **(E)** SCORAD, **(F)** EASI, **(G)** HADS-A, and **(H)** HADS-D.

Subsequently, we investigated the association of *CHN1* and *CXCR6* with the clinical features of AD patients (**Figure 7**). *CHN1* expression positively correlated with SCORAD (r = 0.53, *P* = 0.015), EASI (r = 0.47, *P* = 0.037) and HADS-D (r = 0.56, *P* = 0.011). However, no significant correlation was observed with HADS-A (r = 0.44, *P* = 0.053). In contrast, CXCR6 expression showed no significant correlation with SCORAD (r = -0.28, *P* = 0.238), EASI (r = -0.28, *P* = 0.230), HADS-A (r = -0.17, *P* = 0.474) and HADS-D (r = -0.40, *P* = 0.079). TheX3se findings suggested *CHN1* as a potential biomarker of both lesion severity and psychological burden in AD patients with comorbid depression.

### 3.8 Validation of *CHN1* expression in clinical samples

**Figure 8:**
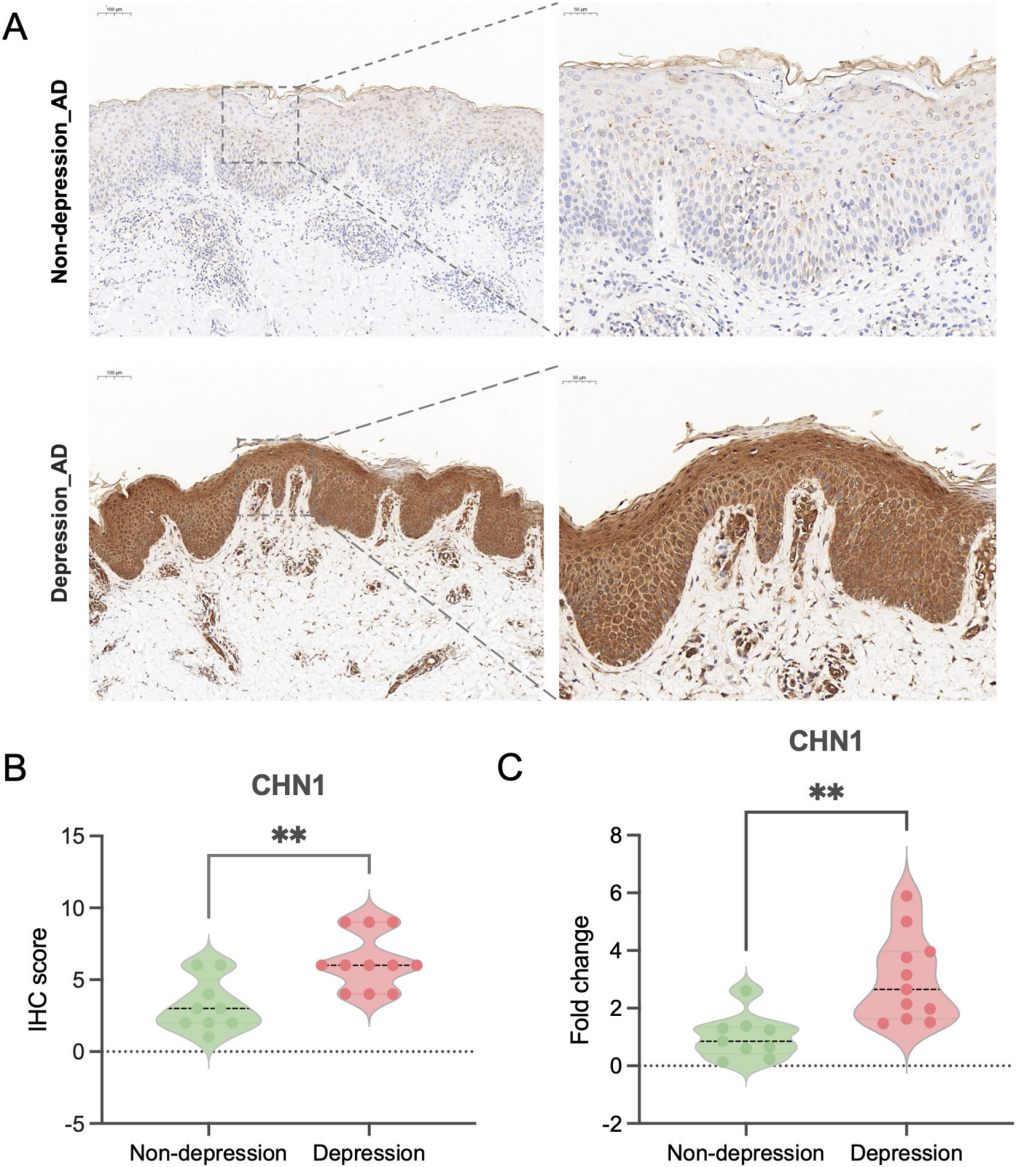
Verification of CHN1 expression in clinical samples. **(A)** IHC staining showing representative CHN1 expression in skin lesions of AD patients. **(B)** Histograms showing IHC scores of CHN1 expression in AD patients with depression (N = 11) and without depression (N = 9). **(C)** qPCR analysis of CHN1 mRNA expression levels in PBMCs from AD patients with (N = 11) and without depression (N = 9). **p < 0.01.

To further verify the correlation between *CHN1* and AD-related depression, qPCR and IHC analysis were performed in samples of AD patients with (N = 11) and without depression (N = 9), with cohort demographics and clinical characteristics detailed in **Table 2**. IHC staining of the skin lesions revealed markedly stronger *CHN1* expression in the lesion tissues of AD patients with depression compared to those without (**Figures 8A, B**). Similarly, qPCR analysis demonstrated significantly higher levels of *CHN1* expression in PBMCs of AD patients with depression (**Figure 8C**). These findings supported the critical role of *CHN1* in the pathogenesis of AD-related depression.

## 4 Discussion

This study firstly combined machine learning techniques with whole-blood transcriptome analysis to explore inflammation-related depression in AD patients. Through these analyses, we identified several key neural and immune pathways enriched in depressed AD, including T-cell receptor signaling, immunoregulatory interactions, negative regulation of Th17 cell differentiation, and neurotransmitter uptake. We further pinpointed *CHN1* as a potential biomarker closely linked to both the inflammatory response and depression severity. These findings suggest that depression in AD may be driven by long-lasting peripheral inflammation influencing central nervous system function.

The comorbidity of AD with anxiety and depression has been observed through large-scale epidemiological and neuroimaging studies (8, 41–43). Skin inflammation activates lesional sensory neurons through cytokine receptors (e.g., IL-31R, IL-4R, etc.) and ion channel receptors (e.g., TRPV1, PAR2, etc.), which transmit signals along a three-neuron pathway to the brain, affecting central itch perception and emotional regulation (44, 45). Chronic pruritus activates brain regions involved in emotion regulation, thereby exacerbating anxiety and depression. A DNFB-induced chronic-pruritus mouse model identified a key anxiety-related circuit- the parabrachial nucleus (PBN) – central medial nucleus of the thalamus (CM) – medial prefrontal cortex (mPFC) pathway (46, 47). Additional mechanisms involving the hippocampus and amygdala dysfunction, further contribute to psychiatric comorbidities (48–50). Notably, chronic skin inflammation also affect the nervous system via the bloodstream, leading to comorbidities across organs and tissues, known as the “atopic march”. For example, cutaneous inflammation in AD can gradually promote the progression of allergic colitis through multiple pathways, including the succinate-Tuft cells-IL-25-ILC2s axis, mitochondrial DNA-STING signaling pathway, and the TSLP-eosinophil axis, which all mediate organ-to-organ communication through the circulation (51, 52). Elevated levels of pro-inflammatory factors in the circulation are likewise common in depressed patients, supporting a mechanistic link between peripheral immune activation and the neuropsychiatric disorders (53, 54). Our findings expand on this concept, suggesting that inflammatory mediators transmitted through the blood circulation may contribute to the development of psychiatric comorbidities in AD.

We further identified *CHN1* as a key gene significantly upregulated in PBMCs of AD patients with depression. Correlation analysis demonstrated a positive association between *CHN1* expression and clinical severity scores, including HADS-D, SCORAD, and EASI scores. *CHN1* encodes a2-chimaerin, a regulator of the Rho GTPase - activating protein (GAP), crucial in neurogenesis and axon guidance (55, 56). As a negative regulator of Rac1 in hippocampal neurons, loss of *CHN1* disrupts dendritic branching and augments poly - innervated spine formation (57, 58). The deficiency of *CHN1* during the embryonic or juvenile period results in a remarkable impact on the cognitive function and behavioral manifestations (59). Moreover, *CHN1* has been implicated in neurodegenerative diseases, such as Alzheimer’s disease and Parkinson’s disease (60, 61), and has shown to have differential expression in inflammatory disorders like asthma (62), psoriasis (63), and dermatomyositis (64), suggesting a role in neuro-immune signaling linking peripheral inflammation to the neural function. In our validation cohort, qPCR and IHC confirm *CHN1* upregulation in both peripheral blood and skin lesions of AD patients with depression. Given prior evidence of neuro-immune circuit engagement in AD, we propose that neuronal *CHN1* elevation may remodel Rac1-dependent axons, heighten pruriceptor excitability, and amplify neuropeptide release that skews cutaneous immunity (65, 66). These results identify *CHN1* as a key neuroimmune mediator and potential biomarker for depression risk in AD. Clinically, *CHN1* assessment could enable (i) the early identification of high-risk patients, (ii) monitoring of symptom burden and therapeutic response, and (iii) development of CHIN1- targeted interventions once mechanistic pathways are validated. Longitudinal and interventional researches are warranted to establish robust assay platforms, clinically meaningful cutoffs, and correlations with standardized psychiatric outcomes.

Despite these advances, several limitations requiring consideration. The modest size of the validation cohort may restrict the statistical power, increase the risk of overfitting and biological uncertainty, common challenges in small-sample WGCNA analysis and machine-learning analyses that can generate overly optimistic performance estimates. Also, age and SCORAD/EASI scores were not modeled as continuous covariates in the RNA-seq analysis, which may have inflated the number of false-positive DEGs. The limitation may restrict the generalizability of our findings and the robustness of validating *CHN1* as a stable biomarker, particularly given the heterogeneity of immune pathway dysregulation among different subgroups of patients with AD and depression comorbidity. Future multi-center studies with larger cohorts are therefore needed to validate the reliability of *CHN1* and refine its clinical applicability. Additionally, mechanistic studies should further elucidate the pathways underlying f neuropsychiatric comorbidities in AD, ultimately guiding targeted therapeutic strategies to improve the mental-health outcomes in AD populations.

## 5 Conclusion

This study advances our understanding of the intricate relationship between AD and depression. By employing bioinformatics and machine learning techniques, we identified *CHN1* as a promising biomarker associated with depression in AD patients. Clinical validation confirmed a marked upregulation of *CHN1* in peripheral blood and its strong correlation with disease-severity indices. Moreover, increased expression of *CHN1* in skin lesions of AD patients with depression underscored *CHN1*’s potential as a biomarker or therapeutic target. Targeting *CHN1* may provide new insights into the neuro-immune mechanisms driving depressive symptoms and pave the way for more precise and effective management of neuropsychiatric comorbid symptoms in AD population.

## Data Availability

The data presented in the study are deposited in the NCBI Gene Expression Omnibus (GEO) repository, accession number GSE307177.

## Supplementary material

Supplementary Figure 1 **(A)**. Venn diagram screening overlapping DEGs identified by different analytical model. **(B)**. Determination of soft-thresholding power in WGCNA. **(C)**. Heatmap of the Topological Overlap Matrix for all genes.
