# Supplementary figures and images for: CHN1 as a potential predictive genetic biomarker for atopic dermatitis-related depression
Source: Front Immunol. 2025 Nov 17;16:1677275. doi: 10.3389/fimmu.2025.1677275 (PMC12665567; doi:10.3389/fimmu.2025.1677275)

## CHN1

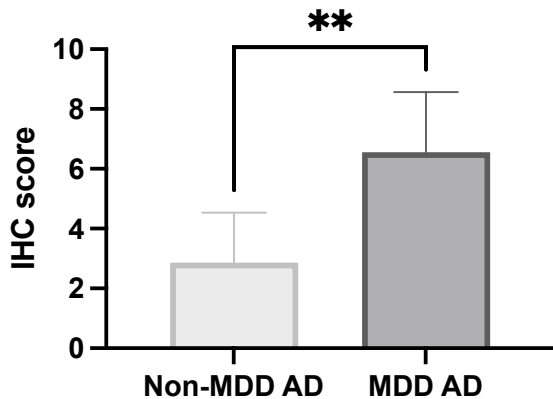

## CHN1

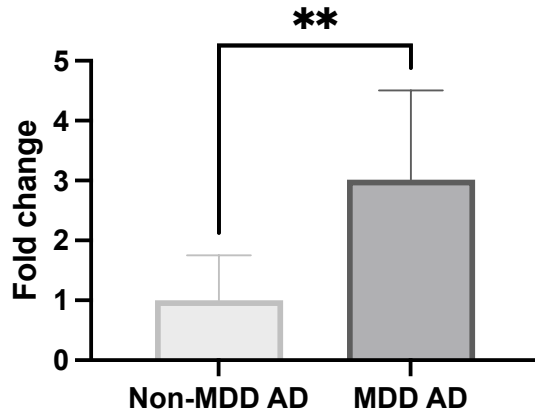

Supplement: Supplementary Figure 1 — (A). Venn diagram screening overlapping DEGs identified by different analytical model. (B). Determination of soft-thresholding power in WGCNA. (C). Heatmap of the Topological Overlap Matrix for all genes. [file DataSheet1.zip › Raw data/CHN1-IHC/Layout 1.pdf]

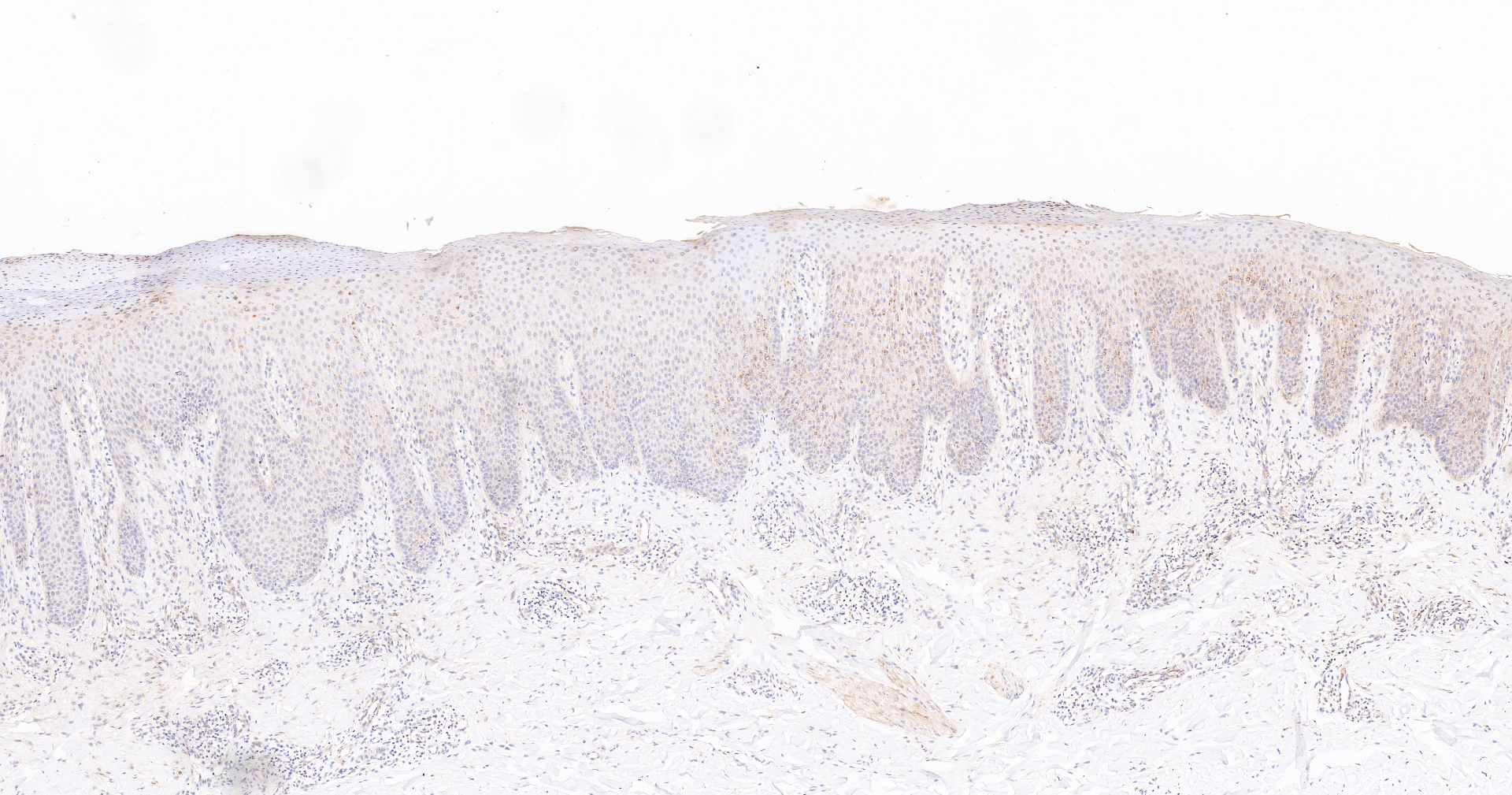

Supplement: Supplementary Figure 1 — (A). Venn diagram screening overlapping DEGs identified by different analytical model. (B). Determination of soft-thresholding power in WGCNA. (C). Heatmap of the Topological Overlap Matrix for all genes. [file DataSheet1.zip › Raw data/CHN1-IHC/NAD-1.jpg]

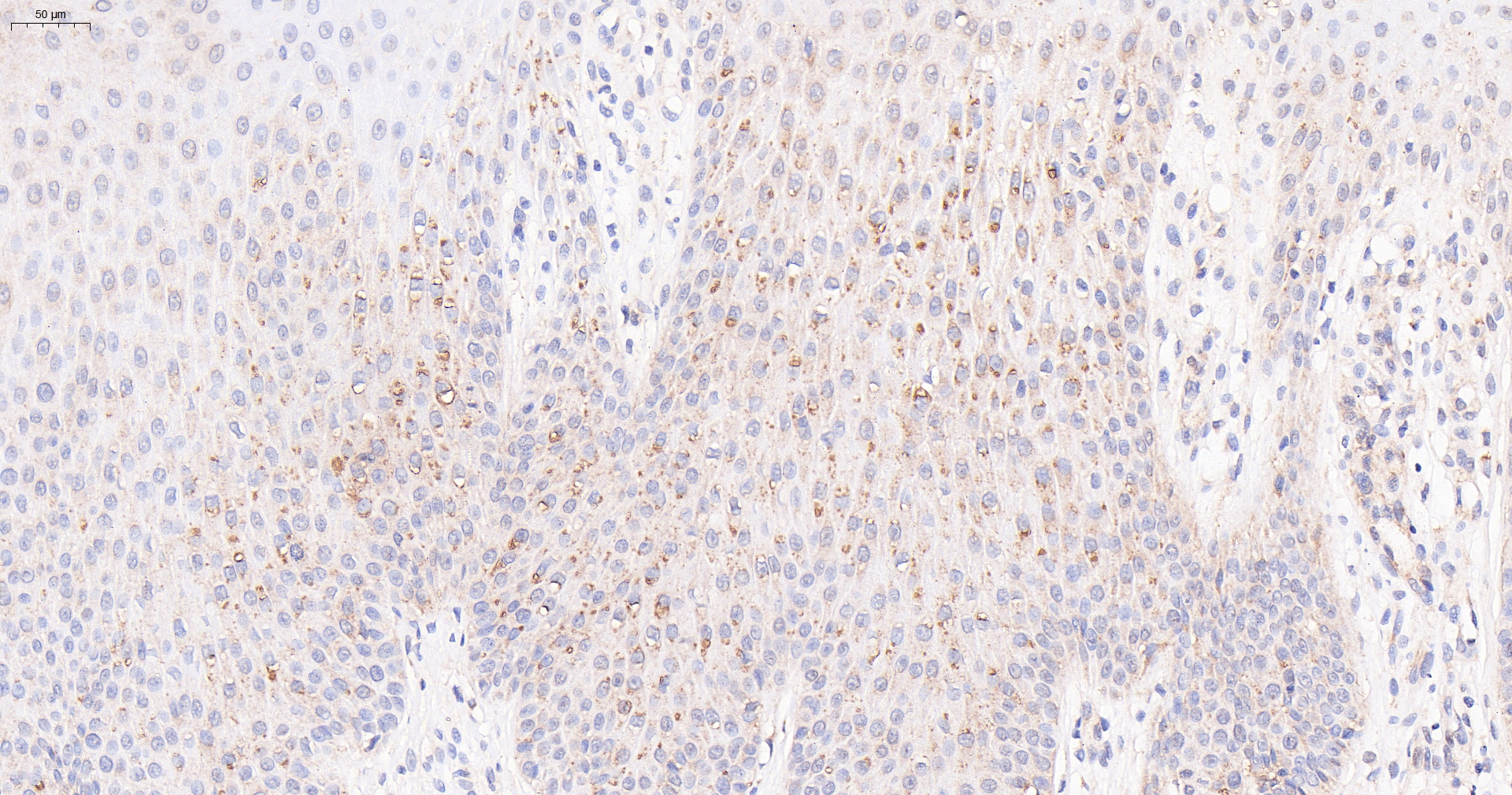

Supplement: Supplementary Figure 1 — (A). Venn diagram screening overlapping DEGs identified by different analytical model. (B). Determination of soft-thresholding power in WGCNA. (C). Heatmap of the Topological Overlap Matrix for all genes. [file DataSheet1.zip › Raw data/CHN1-IHC/NAD-3.jpg]

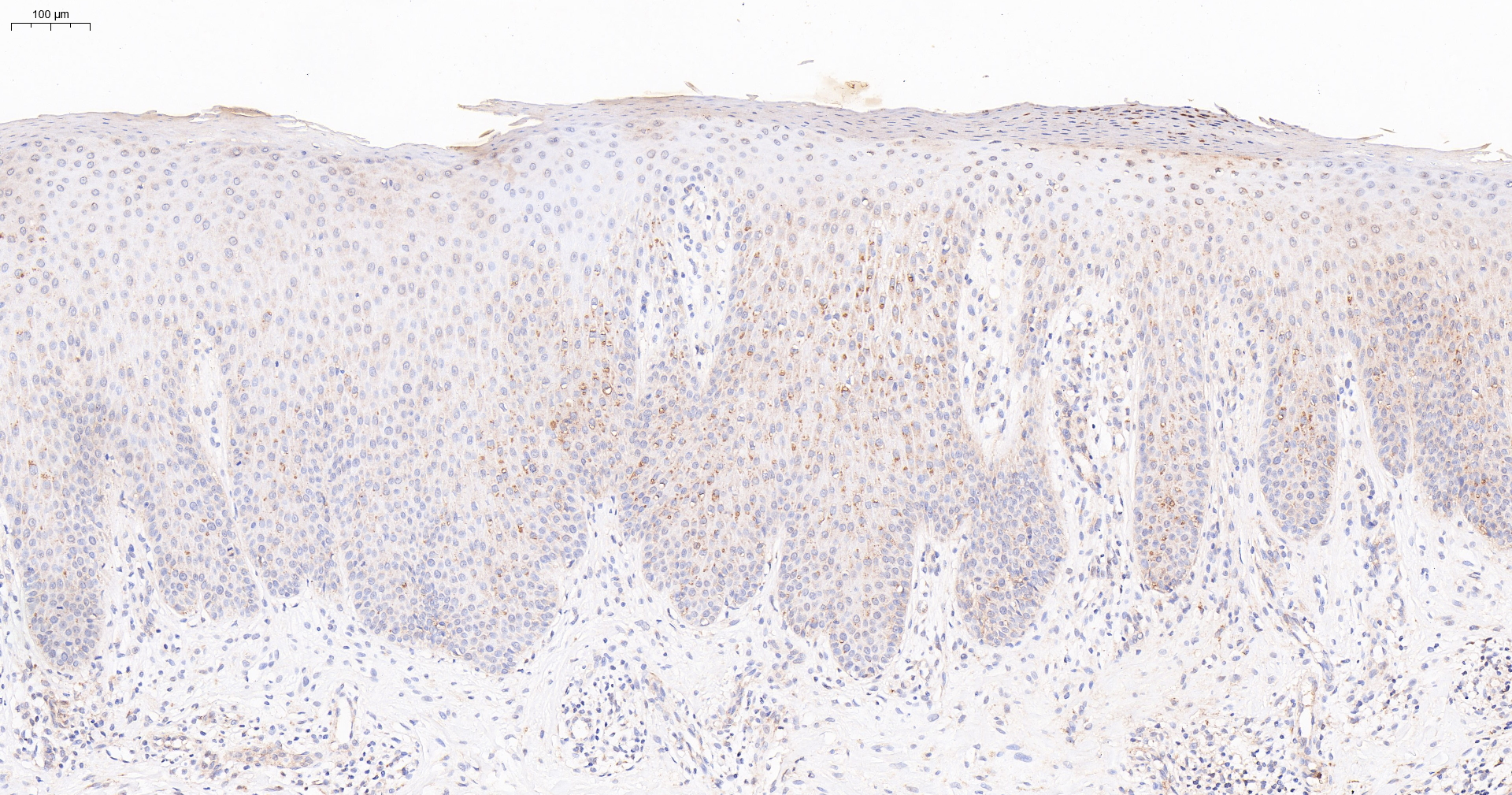

Supplement: Supplementary Figure 1 — (A). Venn diagram screening overlapping DEGs identified by different analytical model. (B). Determination of soft-thresholding power in WGCNA. (C). Heatmap of the Topological Overlap Matrix for all genes. [file DataSheet1.zip › Raw data/CHN1-IHC/NAD-2.jpg]

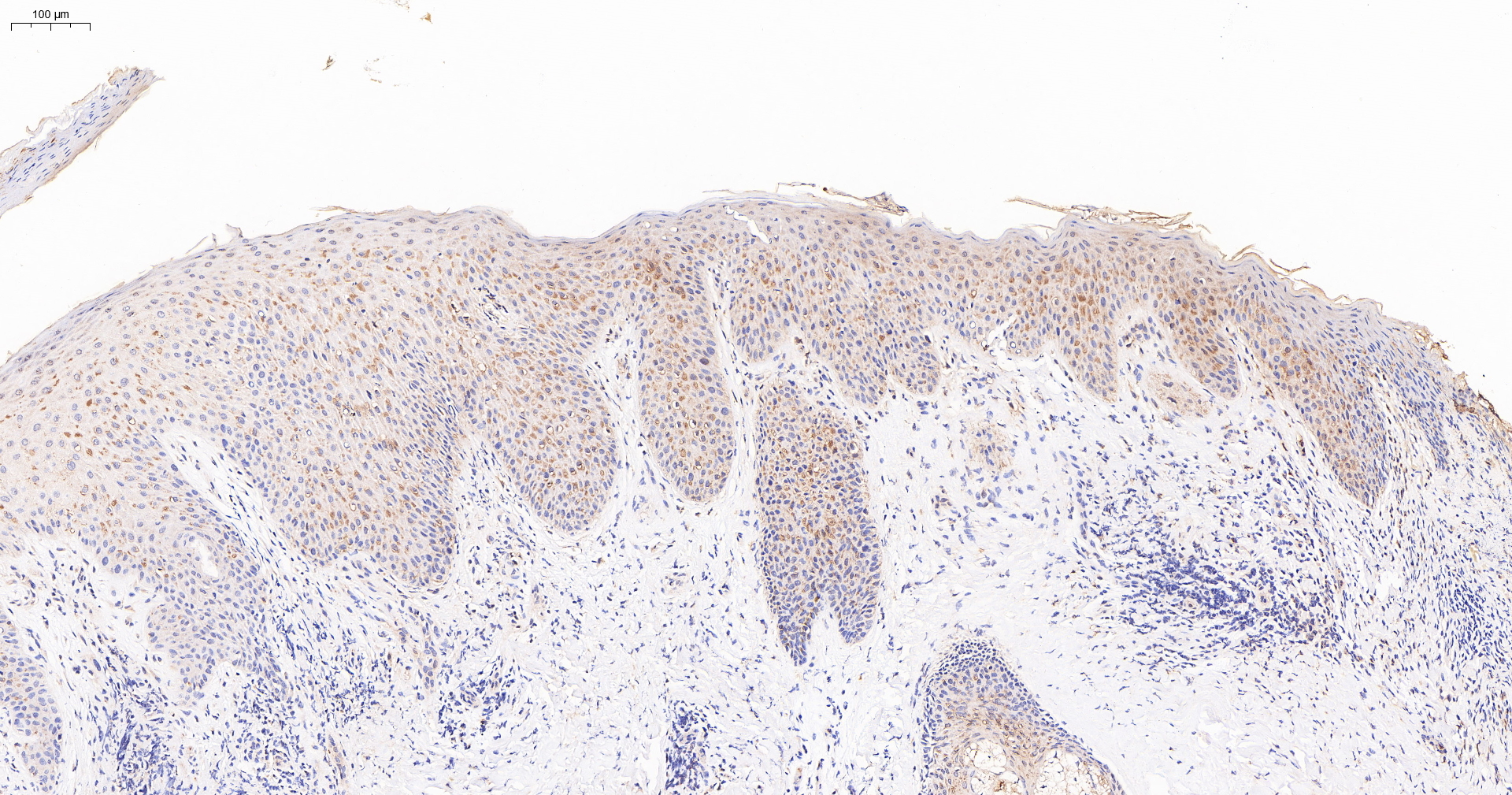

Supplement: Supplementary Figure 1 — (A). Venn diagram screening overlapping DEGs identified by different analytical model. (B). Determination of soft-thresholding power in WGCNA. (C). Heatmap of the Topological Overlap Matrix for all genes. [file DataSheet1.zip › Raw data/CHN1-IHC/NAD-6.jpg]

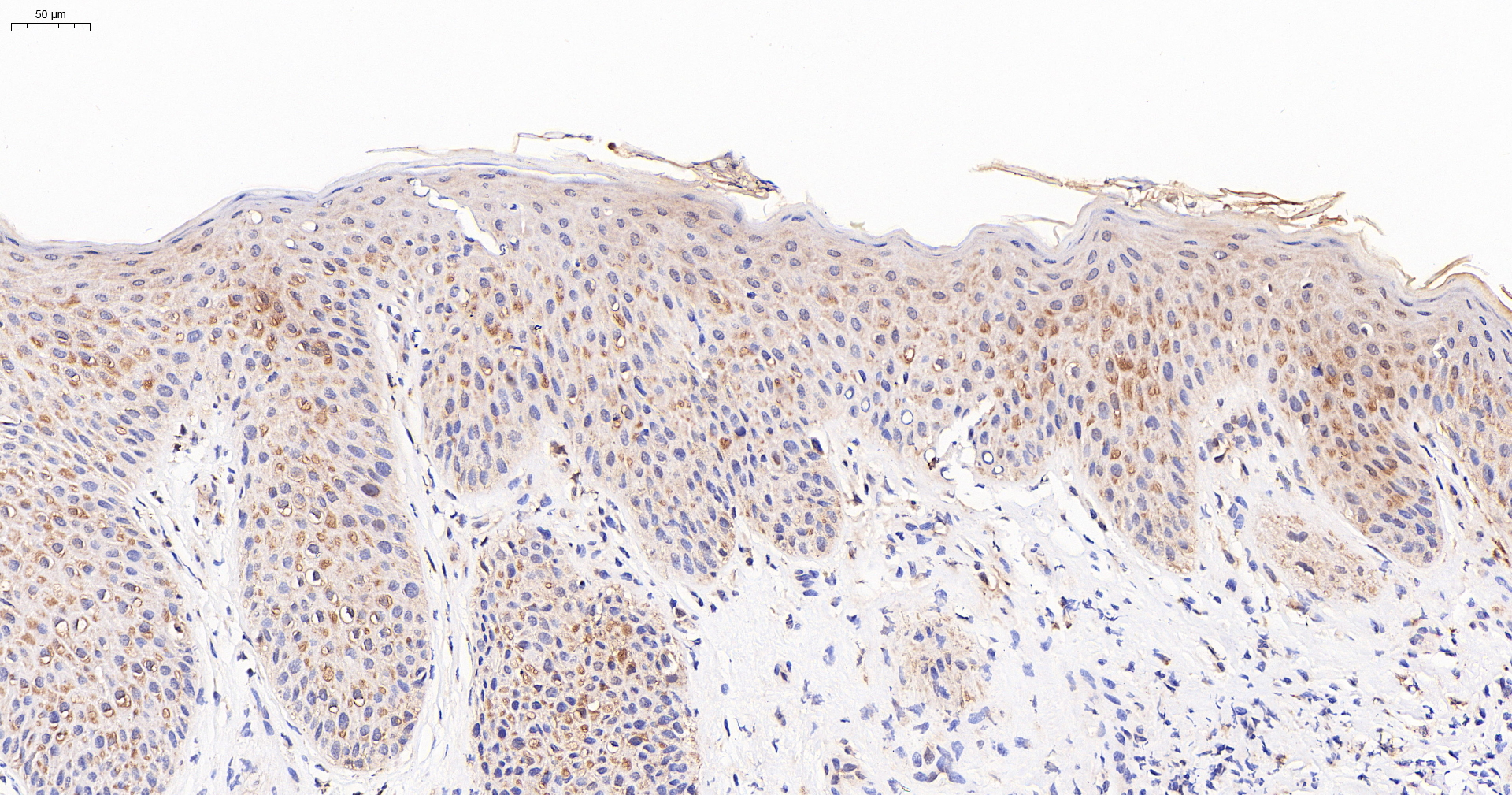

Supplement: Supplementary Figure 1 — (A). Venn diagram screening overlapping DEGs identified by different analytical model. (B). Determination of soft-thresholding power in WGCNA. (C). Heatmap of the Topological Overlap Matrix for all genes. [file DataSheet1.zip › Raw data/CHN1-IHC/NAD-7.jpg]

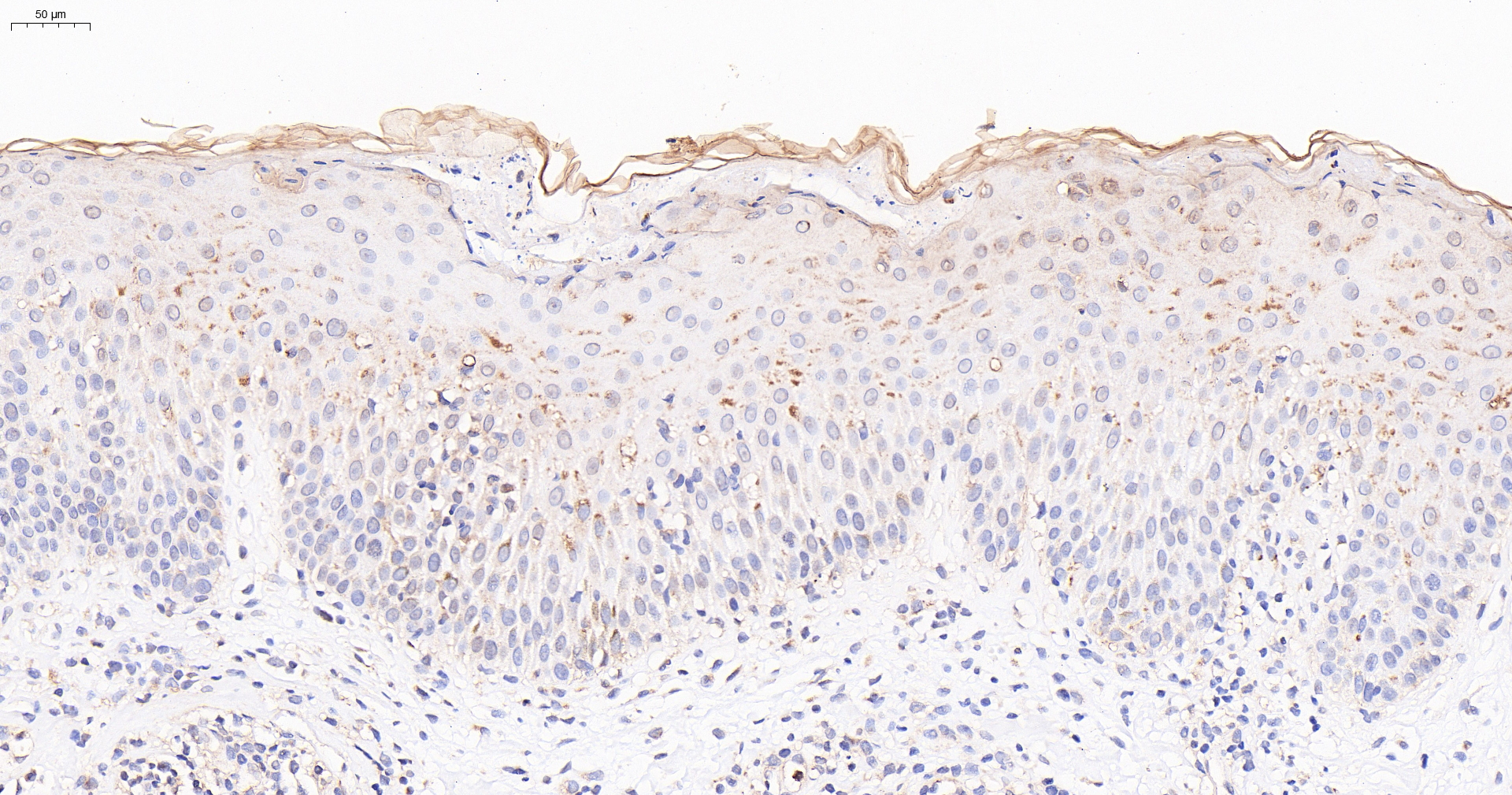

Supplement: Supplementary Figure 1 — (A). Venn diagram screening overlapping DEGs identified by different analytical model. (B). Determination of soft-thresholding power in WGCNA. (C). Heatmap of the Topological Overlap Matrix for all genes. [file DataSheet1.zip › Raw data/CHN1-IHC/NAD-5.jpg]

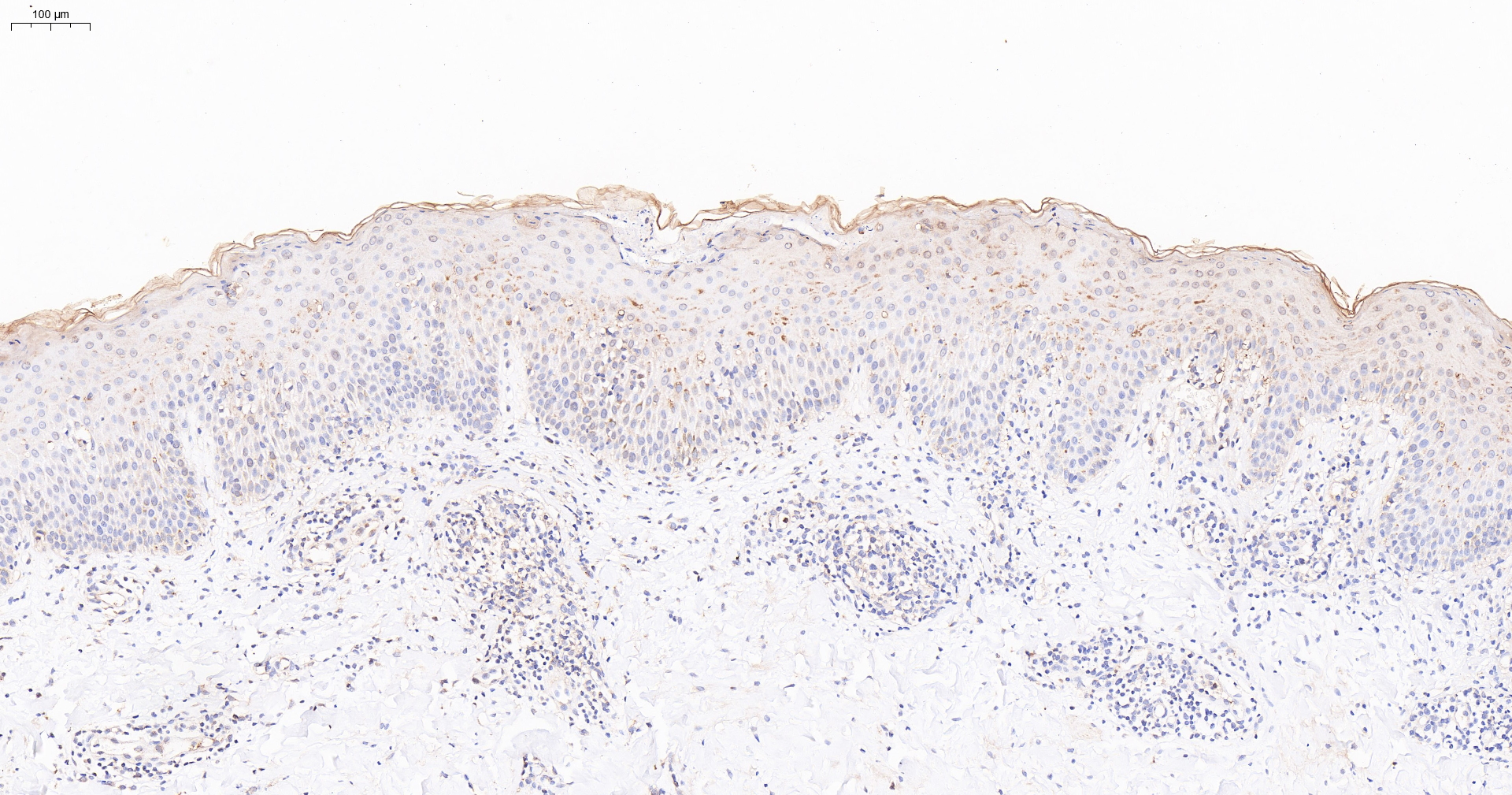

Supplement: Supplementary Figure 1 — (A). Venn diagram screening overlapping DEGs identified by different analytical model. (B). Determination of soft-thresholding power in WGCNA. (C). Heatmap of the Topological Overlap Matrix for all genes. [file DataSheet1.zip › Raw data/CHN1-IHC/NAD-4.jpg]

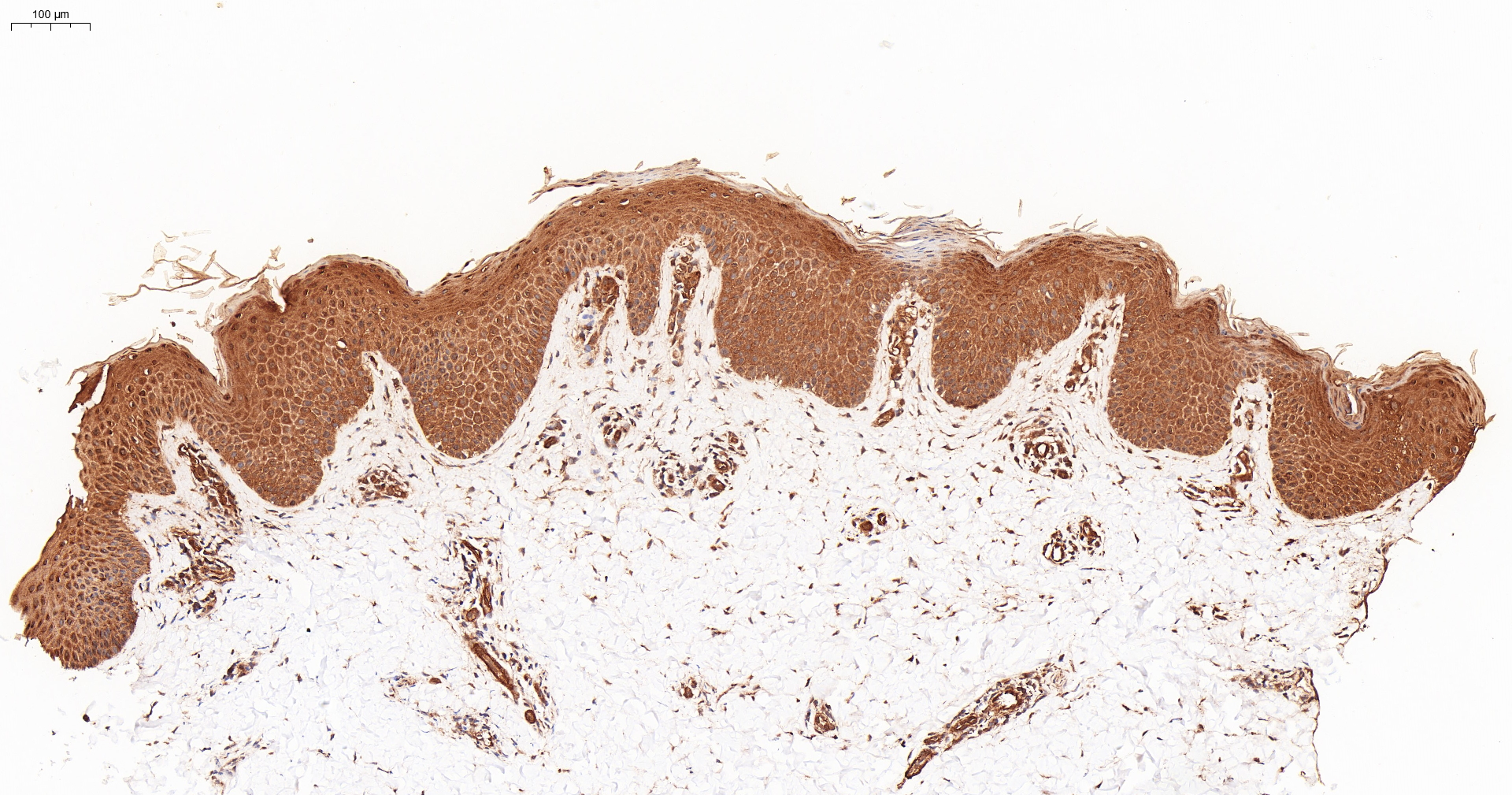

Supplement: Supplementary Figure 1 — (A). Venn diagram screening overlapping DEGs identified by different analytical model. (B). Determination of soft-thresholding power in WGCNA. (C). Heatmap of the Topological Overlap Matrix for all genes. [file DataSheet1.zip › Raw data/CHN1-IHC/DAD-1.jpg]

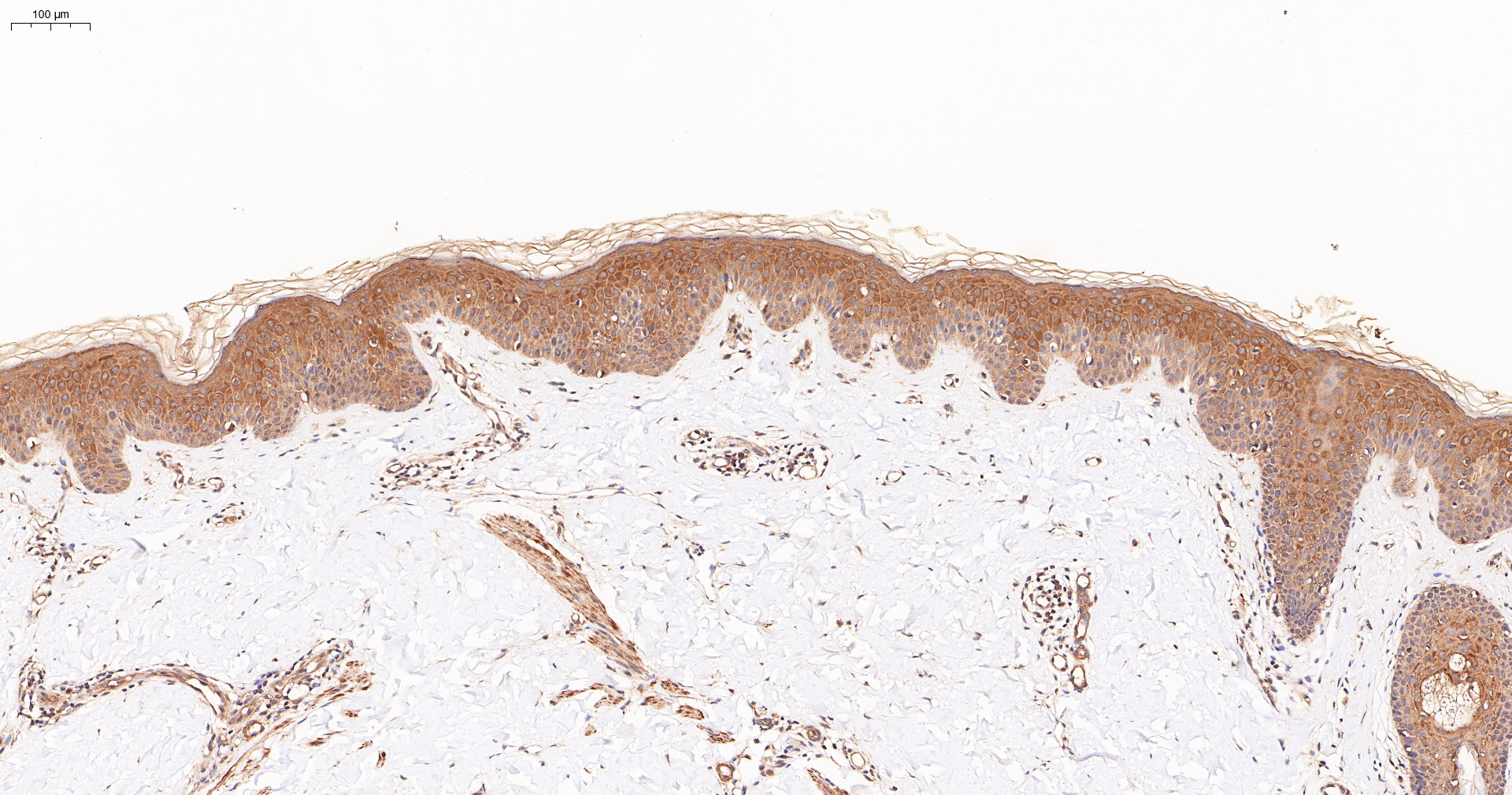

Supplement: Supplementary Figure 1 — (A). Venn diagram screening overlapping DEGs identified by different analytical model. (B). Determination of soft-thresholding power in WGCNA. (C). Heatmap of the Topological Overlap Matrix for all genes. [file DataSheet1.zip › Raw data/CHN1-IHC/DAD-3.jpg]

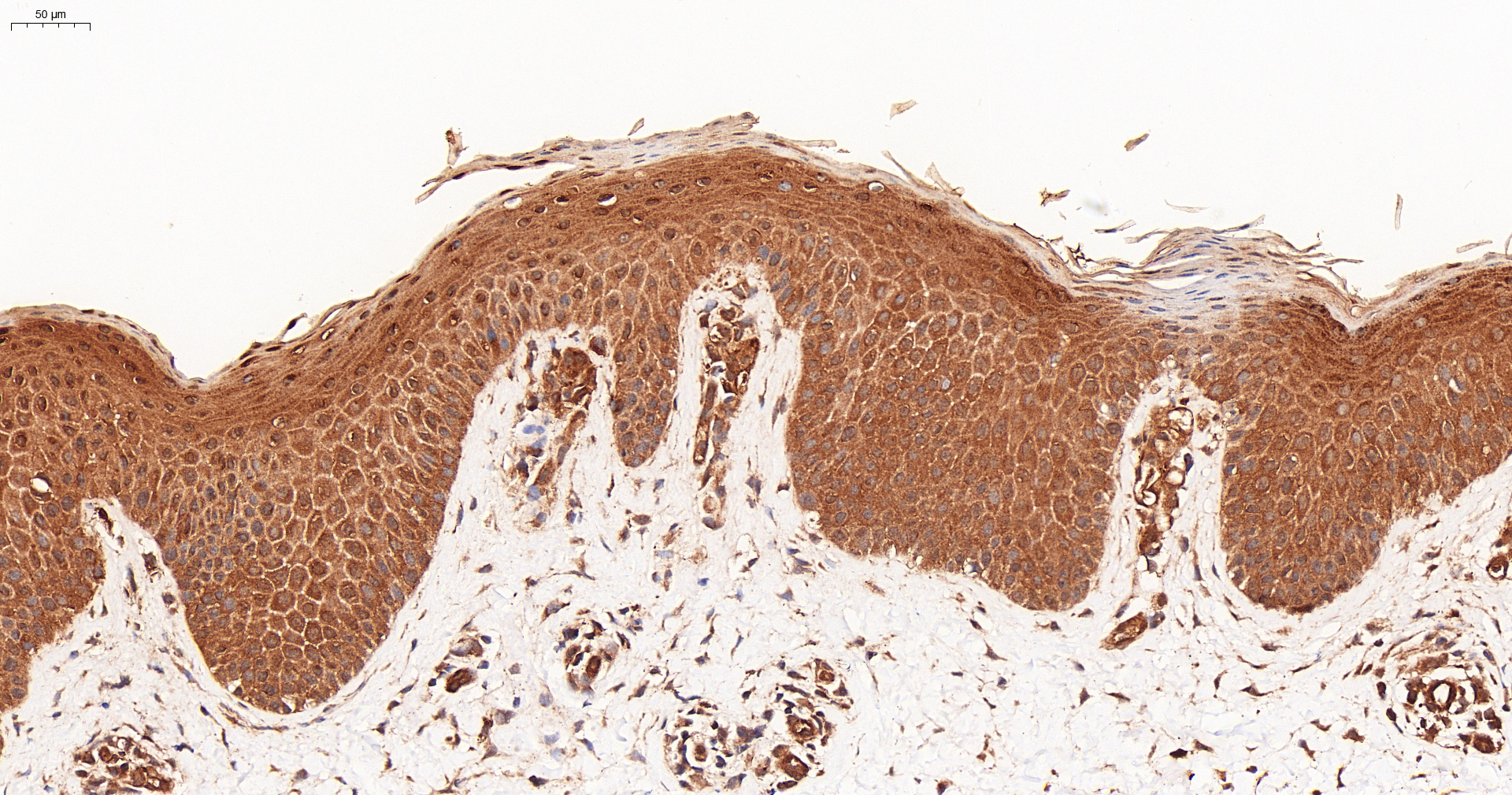

Supplement: Supplementary Figure 1 — (A). Venn diagram screening overlapping DEGs identified by different analytical model. (B). Determination of soft-thresholding power in WGCNA. (C). Heatmap of the Topological Overlap Matrix for all genes. [file DataSheet1.zip › Raw data/CHN1-IHC/DAD-2.jpg]

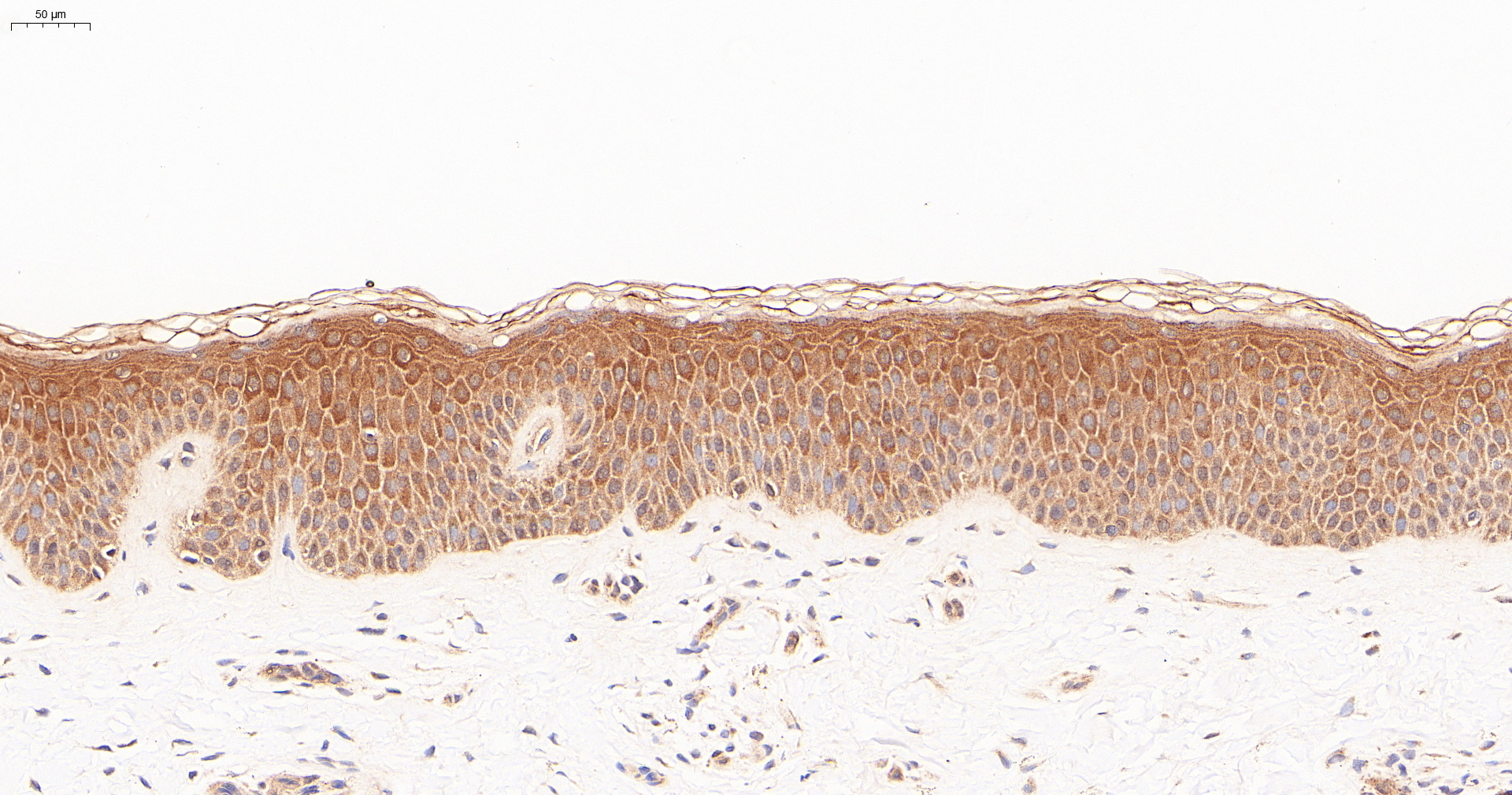

Supplement: Supplementary Figure 1 — (A). Venn diagram screening overlapping DEGs identified by different analytical model. (B). Determination of soft-thresholding power in WGCNA. (C). Heatmap of the Topological Overlap Matrix for all genes. [file DataSheet1.zip › Raw data/CHN1-IHC/DAD-6.jpg]

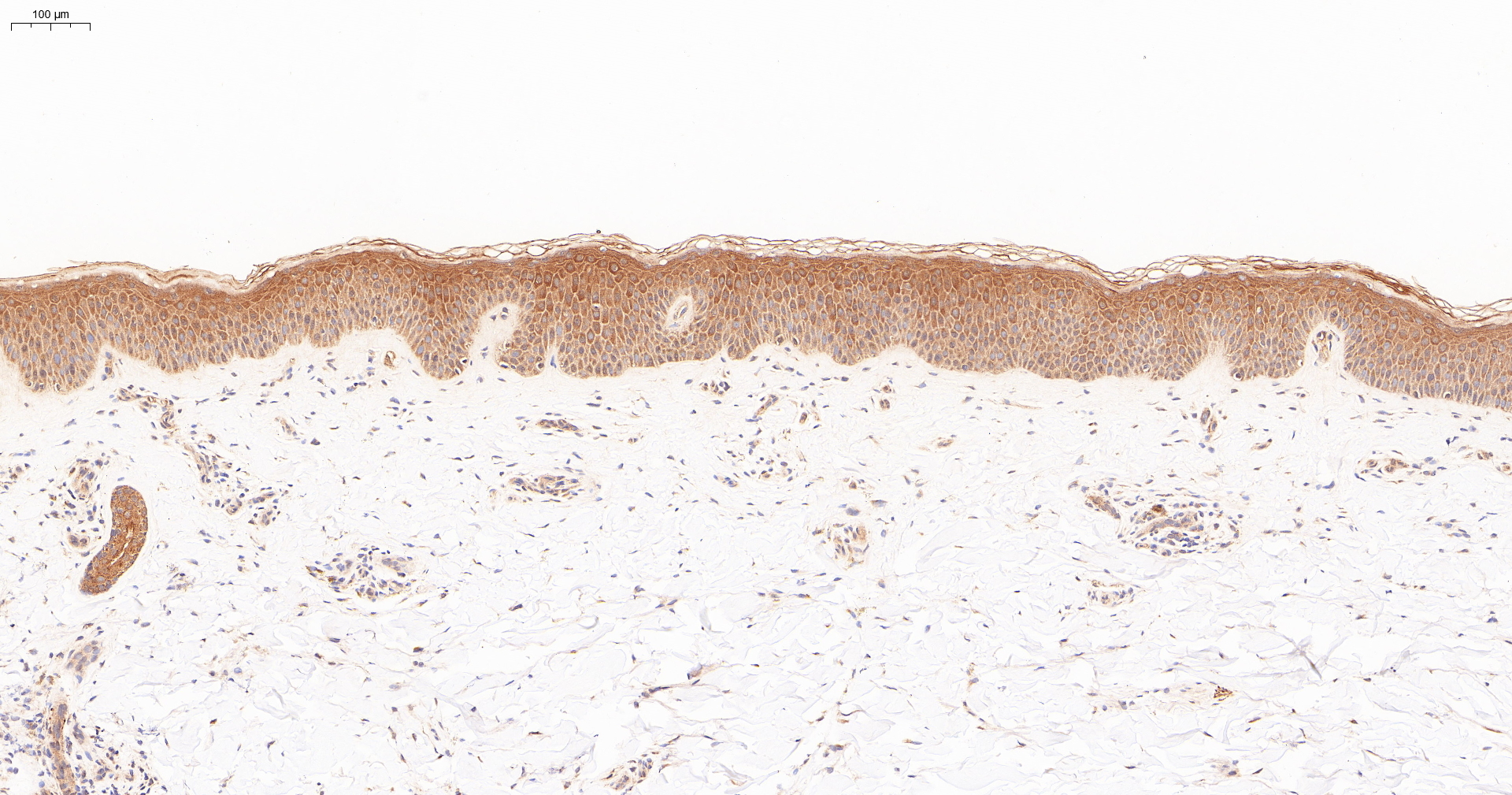

Supplement: Supplementary Figure 1 — (A). Venn diagram screening overlapping DEGs identified by different analytical model. (B). Determination of soft-thresholding power in WGCNA. (C). Heatmap of the Topological Overlap Matrix for all genes. [file DataSheet1.zip › Raw data/CHN1-IHC/DAD-5.jpg]

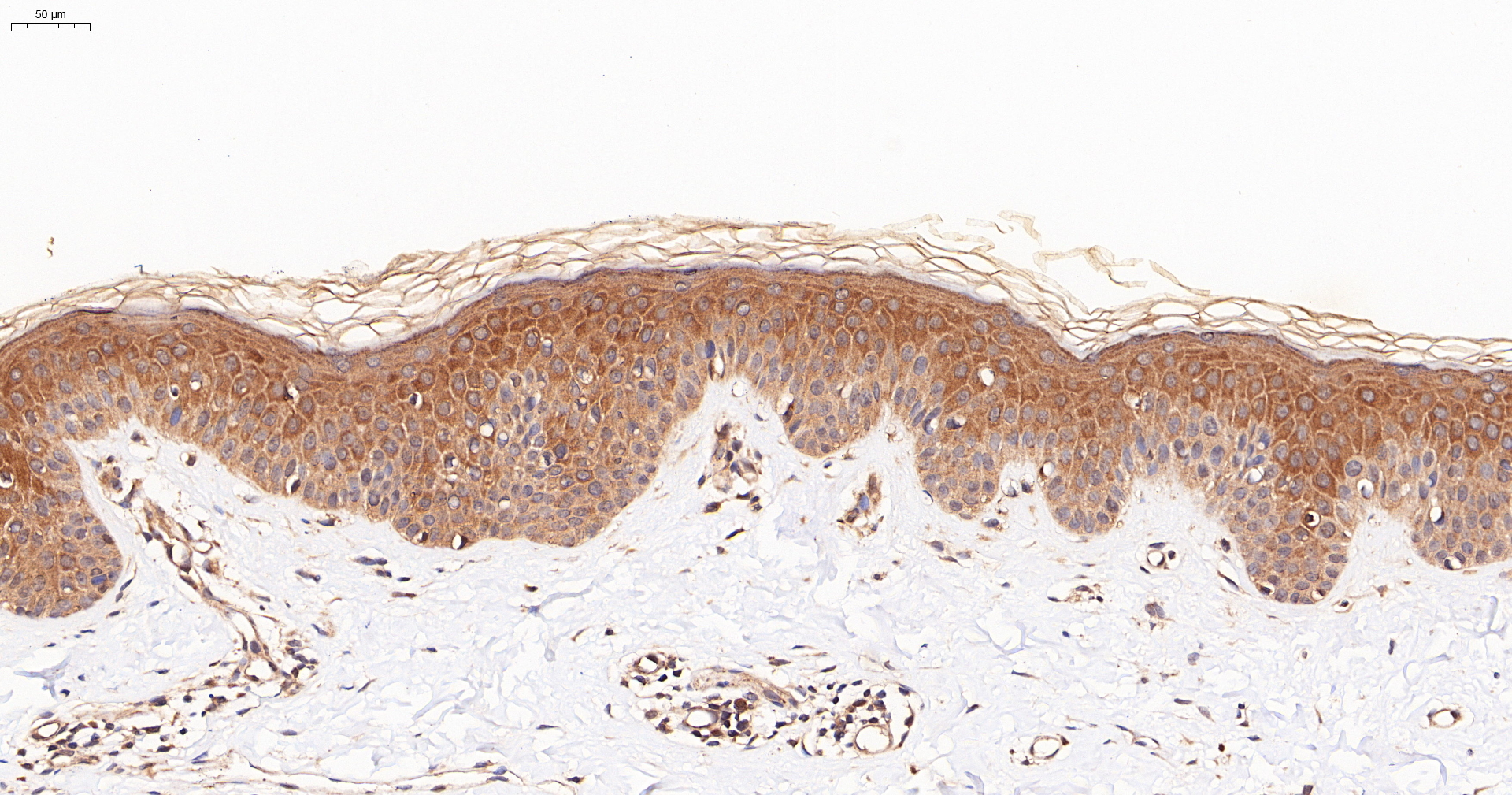

Supplement: Supplementary Figure 1 — (A). Venn diagram screening overlapping DEGs identified by different analytical model. (B). Determination of soft-thresholding power in WGCNA. (C). Heatmap of the Topological Overlap Matrix for all genes. [file DataSheet1.zip › Raw data/CHN1-IHC/DAD-4.jpg]
