# Supplementary material for: CHN1 as a potential predictive genetic biomarker for atopic dermatitis-related depression
Source: Front Immunol. 2025 Nov 17;16:1677275. doi: 10.3389/fimmu.2025.1677275 (PMC12665567; doi:10.3389/fimmu.2025.1677275)
Supplement: Supplementary file 2 [file DataSheet2.pdf]

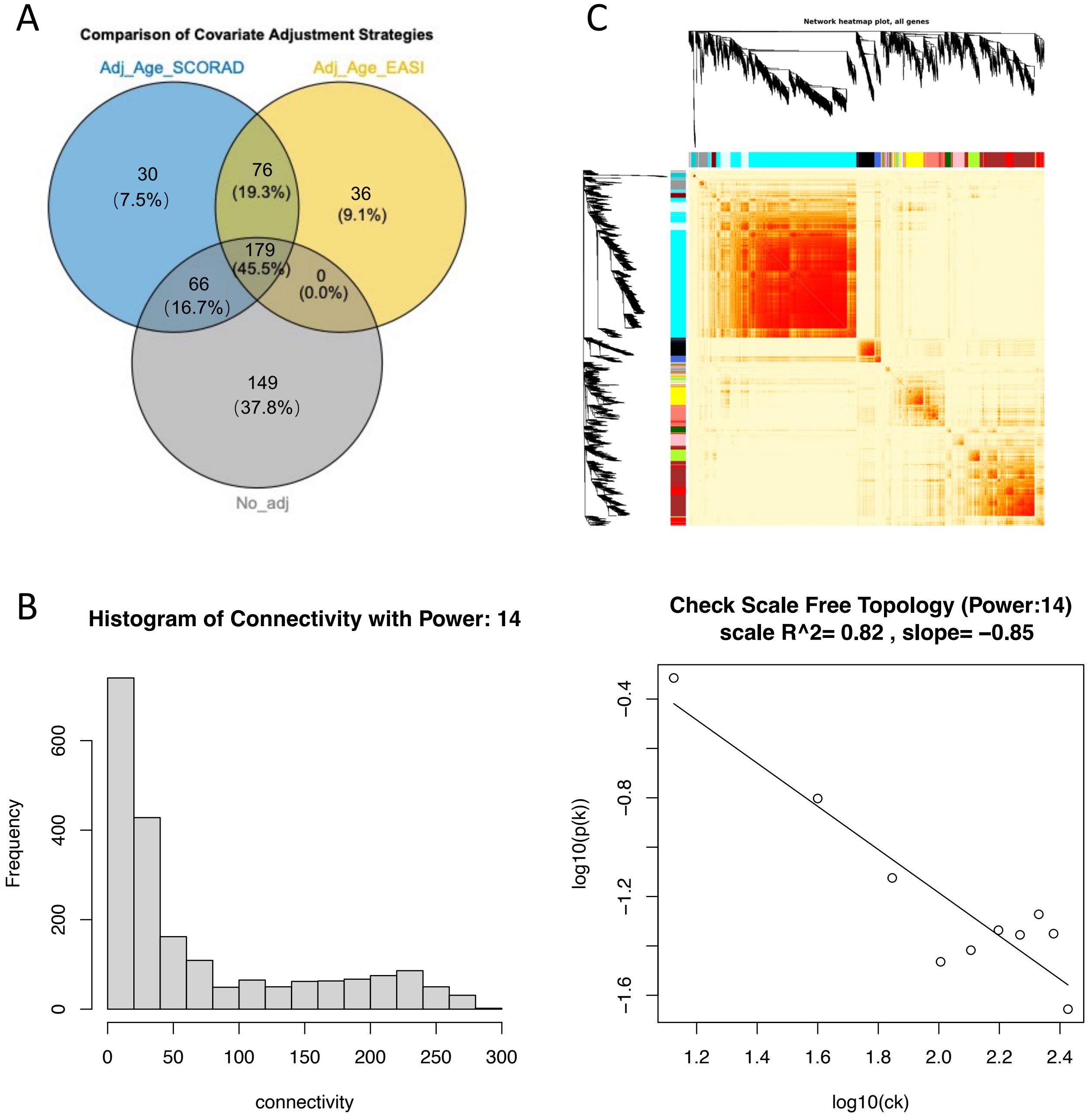
A
Comparison of Covariate Adjustment Strategies
Adj_Age_SCORAD
Adj_Age_EASI
30
(7.5%)
76
(19.3%)
36
(9.1%)
179
(45.5%)
66
(16.7%)
0
(0.0%)
149
(37.8%)
No_adj
C
Network heatmap plot, all genes
B
Histogram of Connectivity with Power: 14
Frequency
0
200
400
600
0
50
100
150
200
250
300
connectivity
Check Scale Free Topology (Power:14)
scale R^2= 0.82 , slope= −0.85
log10(p(k))
−0.4
−0.8
−1.2
−1.6
1.2
1.4
1.6
1.8
2.0
2.2
2.4
log10(ck)
