# Supplementary material for: CHN1 as a potential predictive genetic biomarker for atopic dermatitis-related depression
Source: Front Immunol. 2025 Nov 17;16:1677275. doi: 10.3389/fimmu.2025.1677275 (PMC12665567; doi:10.3389/fimmu.2025.1677275)
Supplement: Supplementary file 3 [file Table1.docx]

Supplementary table 1. Diagnostic Criteria of Depression

| **Diagnostic Criteria of Major Depressive Disorder** | |
| --- | --- |
| **A** | Five (or more) of the following symptoms have been present during the same 2-week period and represent a change from previous functioning; at least one of the symptoms is either (1) depressed mood or (2) loss of interest or pleasure. |
|  | 1. Depressed mood most of the day, nearly every day, as indicated by either subjective report or observation made by others.  2. Markedly diminished interest or pleasure in all, or almost all, activities most of the day, nearly every day.  3. Significant weight loss when not dieting or weight gain, or decrease or increase in appetite nearly every day.  4. Insomnia or hypersomnia nearly every day.  5. Psychomotor agitation or retardation nearly every day.  6. Fatigue or loss of energy nearly every day.  7. Feelings of worthlessness or excessive or inappropriate guilt nearly every day.  8. Diminished ability to think or concentrate, or indecisiveness, nearly every day.  9. Recurrent thoughts of death, recurrent suicidal ideation without a specific plan, or a suicide attempt or a specific plan for committing suicide. |
| **B** | The symptoms cause clinically significant distress or impairment in social, occupational, or other important areas of functioning. |
| **C** | The episode is not attributable to the physiological effects of a substance or another medical condition. |
| **D** | At least one major depressive episode is not better explained by schizoaffective disorder and is not superimposed on schizophrenia, schizophreniform disorder, delusional disorder, or other specified and unspecified schizophrenia spectrum and other psychotic disorders. |
| **E** | There has never been a manic episode or a hypomanic episode. |

| **Diagnostic Criteria of Persistent Depressive Disorder** | |
| --- | --- |
| **A** | Depressed mood for most of the day, for more days than not, as indicated by either subjective account or observation by others, for at least 2 years. |
| **B** | Presence, while depressed, of two (or more) of the following: |
|  | 1. Poor appetite or overeating.  2. Insomnia or hypersomnia.  3. Low energy or fatigue.  4. Low self-esteem.  5. Poor concentration or difficulty making decisions.  6. Feelings of hopelessness. |
| **C** | During the 2-year period (1 year for children or adolescents) of the disturbance, the individual has never been without the symptoms in Criteria A and B for more than 2 months at a time. |
| **D** | Criteria for a major depressive disorder may be continuously present for 2 years. |
| **E** | There has never been a manic episode or a hypomanic episode. |
| **F** | The disturbance is not better explained by a persistent schizoaffective disorder, schizophrenia, delusional disorder, or other specified or unspecified schizophrenia spectrum and other psychotic disorder. |
| **G** | The symptoms are not attributable to the physiological effects of a substance or another medical condition. |
| **H** | The symptoms cause clinically significant distress or impairment in social, occupational, or other important areas of functioning. |
